# Supplementary material for: Accelerated construction of an in vitro model of human periodontal ligament tissue: vacuum plasma combined with fibronectin coating and a polydimethylsiloxane matrix
Source: PeerJ. 2019 May 31;7:e7036. doi: 10.7717/peerj.7036 (PMC6546080; doi:10.7717/peerj.7036)
Supplement: Figure S2 — Atomic force microscopy (AFM, SPM-9600, Shimadzu, Kyoto, Japan) raw data and its translation into English where there are Japanese words. [file peerj-07-7036-s002.zip › Fig2 AFM/Translation from Japanese to English.docx]

| **Japanese** | **English** |
| --- | --- |
| 全体 | Total |
| 長さX | Length X |
| 長さY | Length Y |
| 面積 | Surface Area |
