# Supplementary figures and images for: Accelerated construction of an in vitro model of human periodontal ligament tissue: vacuum plasma combined with fibronectin coating and a polydimethylsiloxane matrix
Source: PeerJ. 2019 May 31;7:e7036. doi: 10.7717/peerj.7036 (PMC6546080; doi:10.7717/peerj.7036)

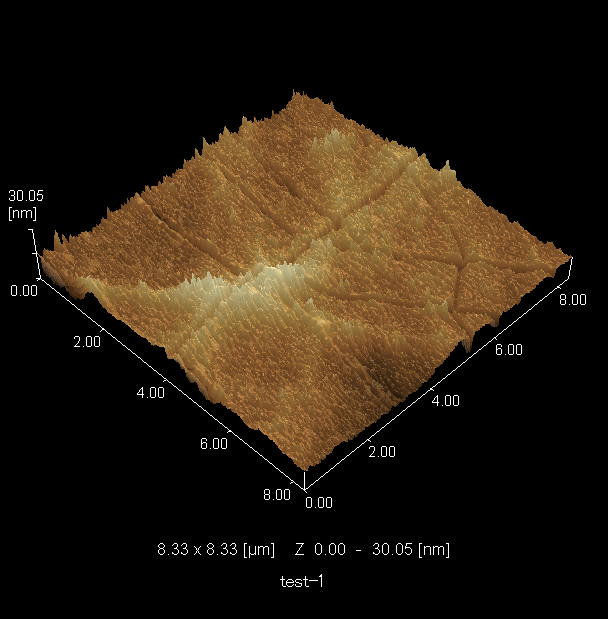

Supplement: Figure S2 — Atomic force microscopy (AFM, SPM-9600, Shimadzu, Kyoto, Japan) raw data and its translation into English where there are Japanese words. [file peerj-07-7036-s002.zip › Fig2 AFM/FN-3D.TIF]

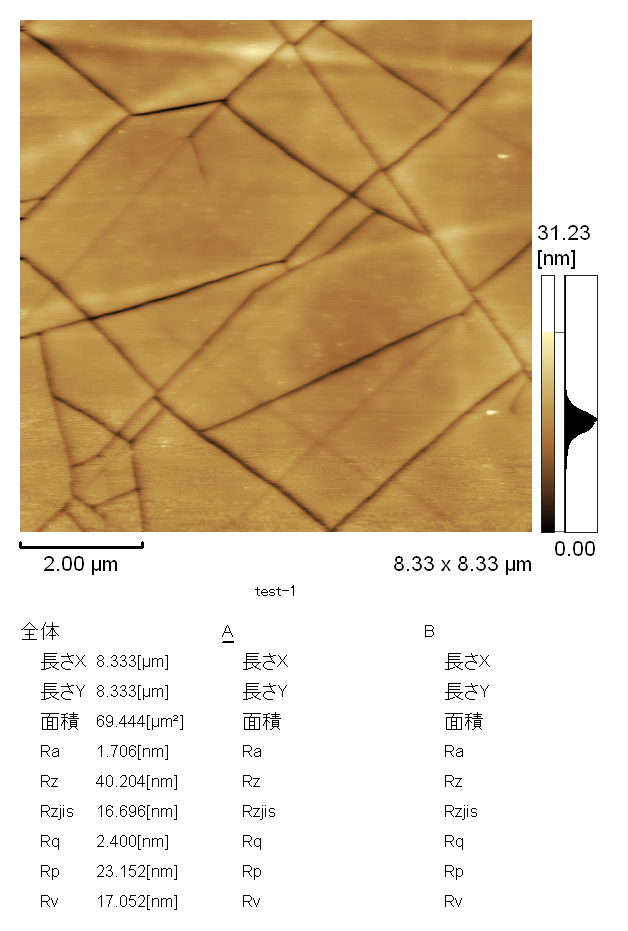

Supplement: Figure S2 — Atomic force microscopy (AFM, SPM-9600, Shimadzu, Kyoto, Japan) raw data and its translation into English where there are Japanese words. [file peerj-07-7036-s002.zip › Fig2 AFM/FN-surface.TIF]

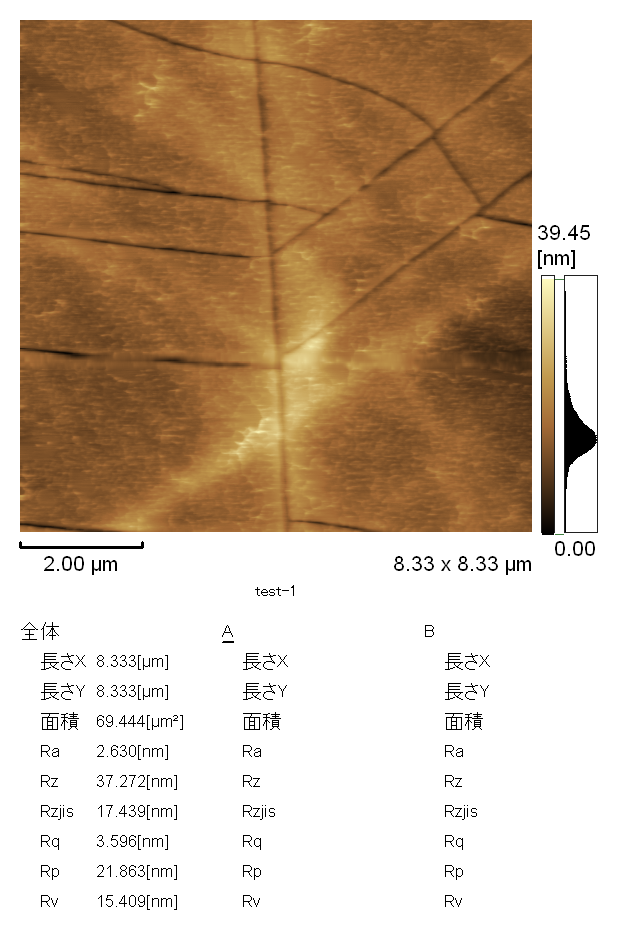

Supplement: Figure S2 — Atomic force microscopy (AFM, SPM-9600, Shimadzu, Kyoto, Japan) raw data and its translation into English where there are Japanese words. [file peerj-07-7036-s002.zip › Fig2 AFM/FN-surface1.TIF]

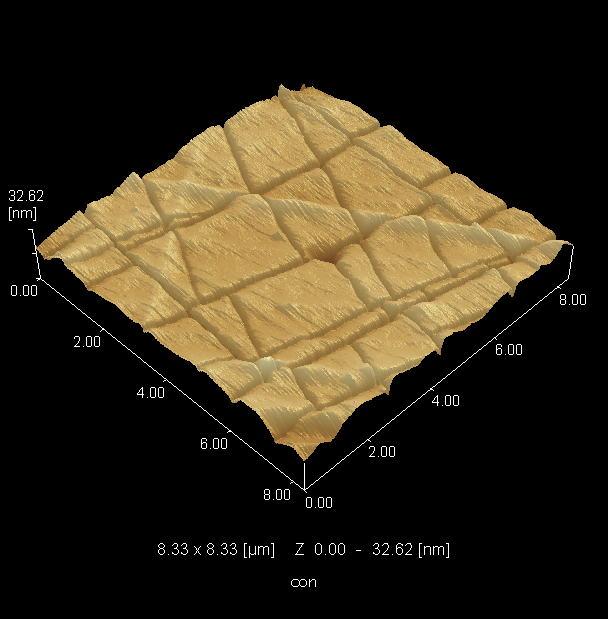

Supplement: Figure S2 — Atomic force microscopy (AFM, SPM-9600, Shimadzu, Kyoto, Japan) raw data and its translation into English where there are Japanese words. [file peerj-07-7036-s002.zip › Fig2 AFM/NN-3D.TIF]

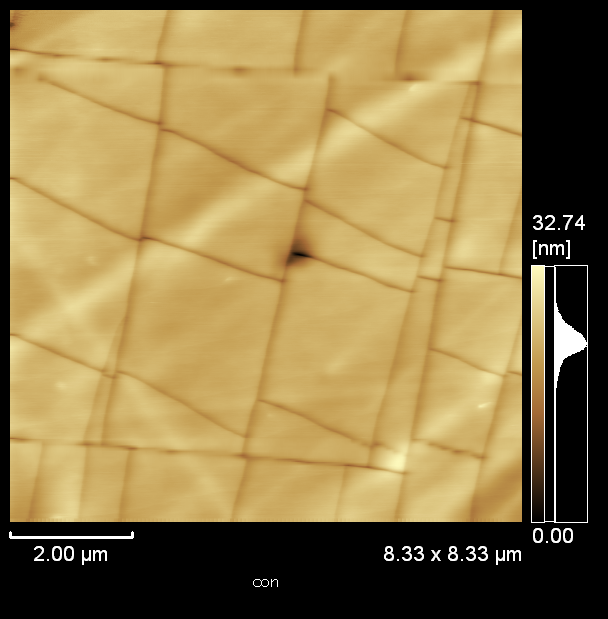

Supplement: Figure S2 — Atomic force microscopy (AFM, SPM-9600, Shimadzu, Kyoto, Japan) raw data and its translation into English where there are Japanese words. [file peerj-07-7036-s002.zip › Fig2 AFM/NN-surface1.TIF]

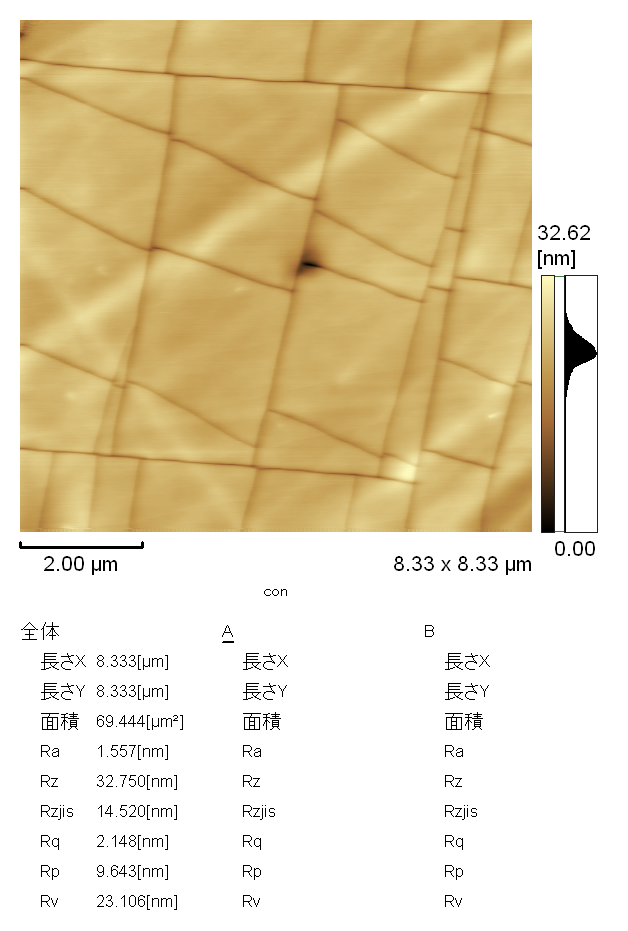

Supplement: Figure S2 — Atomic force microscopy (AFM, SPM-9600, Shimadzu, Kyoto, Japan) raw data and its translation into English where there are Japanese words. [file peerj-07-7036-s002.zip › Fig2 AFM/NN-surface2.TIF]

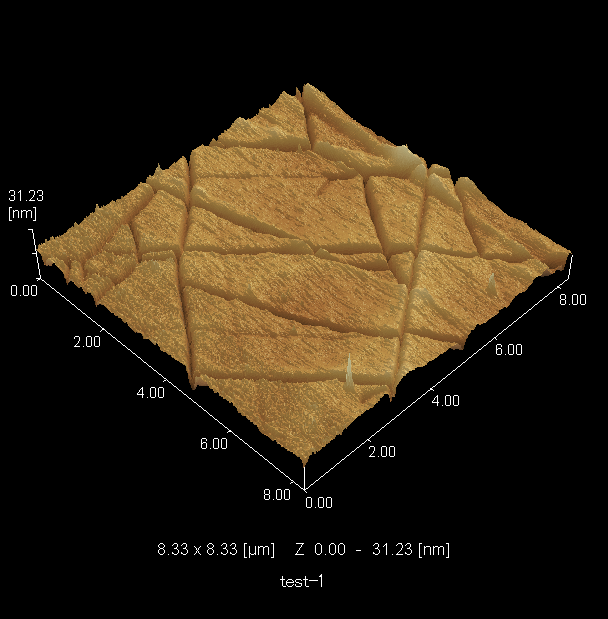

Supplement: Figure S2 — Atomic force microscopy (AFM, SPM-9600, Shimadzu, Kyoto, Japan) raw data and its translation into English where there are Japanese words. [file peerj-07-7036-s002.zip › Fig2 AFM/PF-3D.TIF]

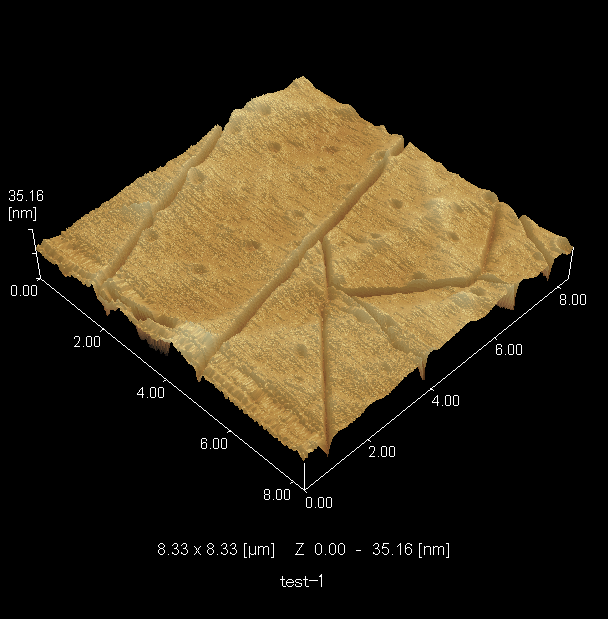

Supplement: Figure S2 — Atomic force microscopy (AFM, SPM-9600, Shimadzu, Kyoto, Japan) raw data and its translation into English where there are Japanese words. [file peerj-07-7036-s002.zip › Fig2 AFM/PN-3D.TIF]

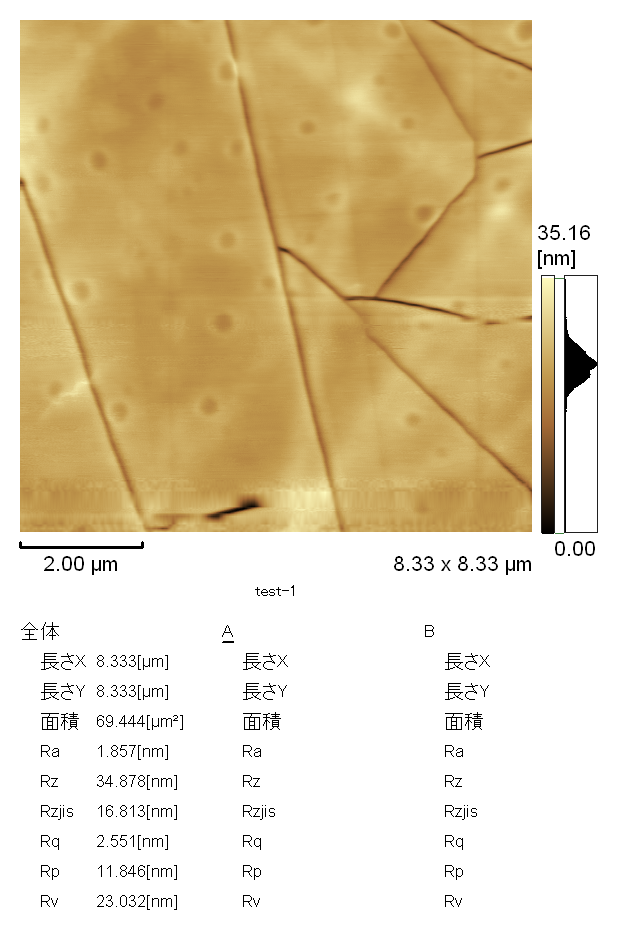

Supplement: Figure S2 — Atomic force microscopy (AFM, SPM-9600, Shimadzu, Kyoto, Japan) raw data and its translation into English where there are Japanese words. [file peerj-07-7036-s002.zip › Fig2 AFM/PN-surface.TIF]

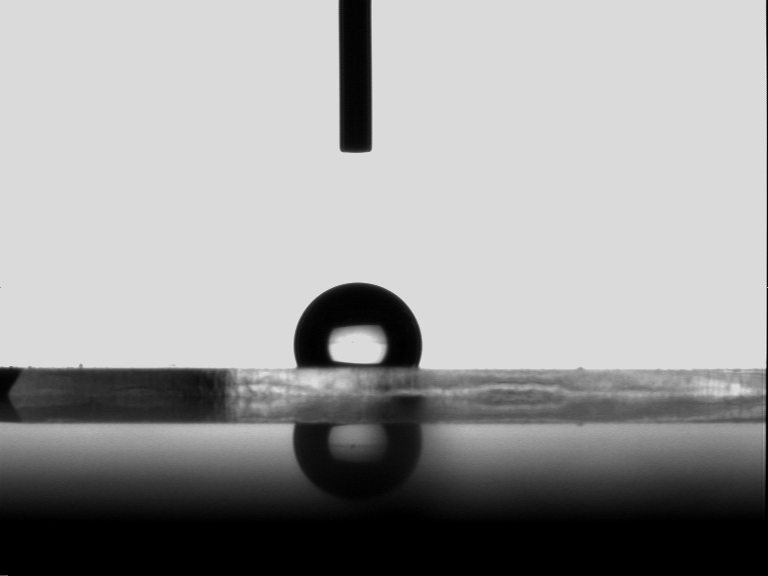

Supplement: Figure S3 — Raw data of wettability measurement using drop-shape analysis (DSA) system (DSA10Mk2, KRÜSS GmbH, Hamburg, Germany). [file peerj-07-7036-s003.zip › Fig3 Wettability/FN.bmp]

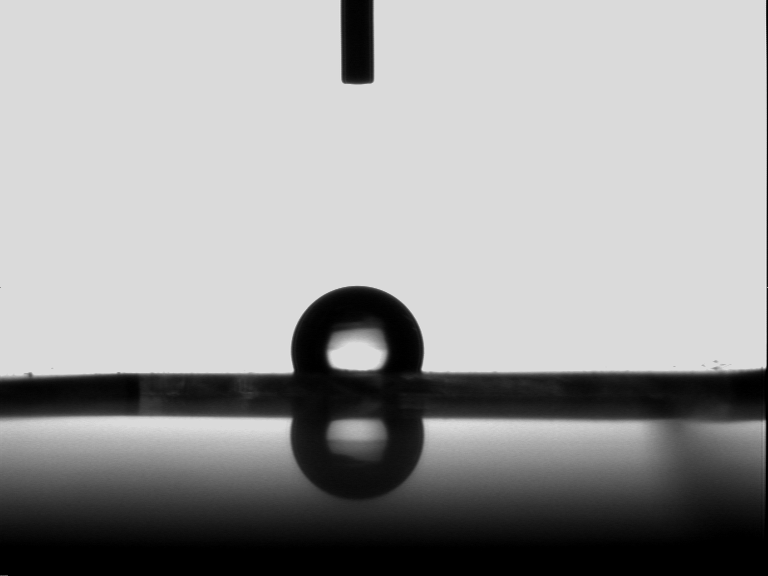

Supplement: Figure S3 — Raw data of wettability measurement using drop-shape analysis (DSA) system (DSA10Mk2, KRÜSS GmbH, Hamburg, Germany). [file peerj-07-7036-s003.zip › Fig3 Wettability/NN.bmp]

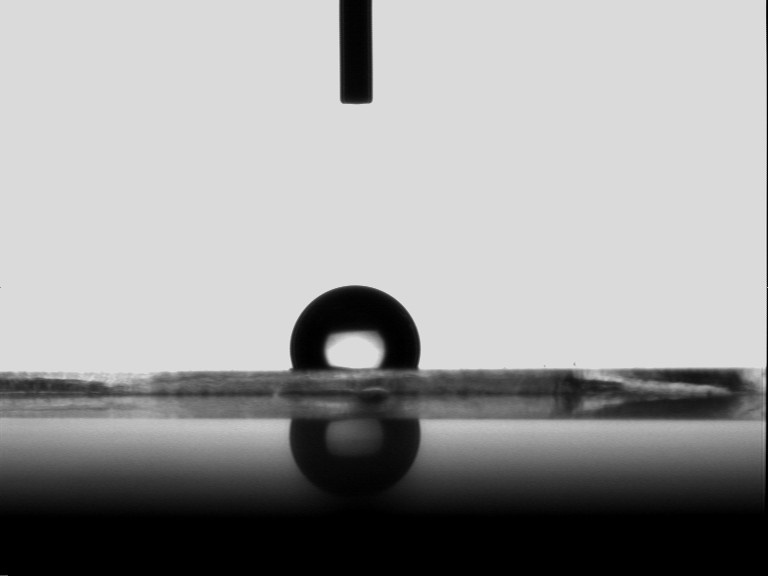

Supplement: Figure S3 — Raw data of wettability measurement using drop-shape analysis (DSA) system (DSA10Mk2, KRÜSS GmbH, Hamburg, Germany). [file peerj-07-7036-s003.zip › Fig3 Wettability/PF.bmp]

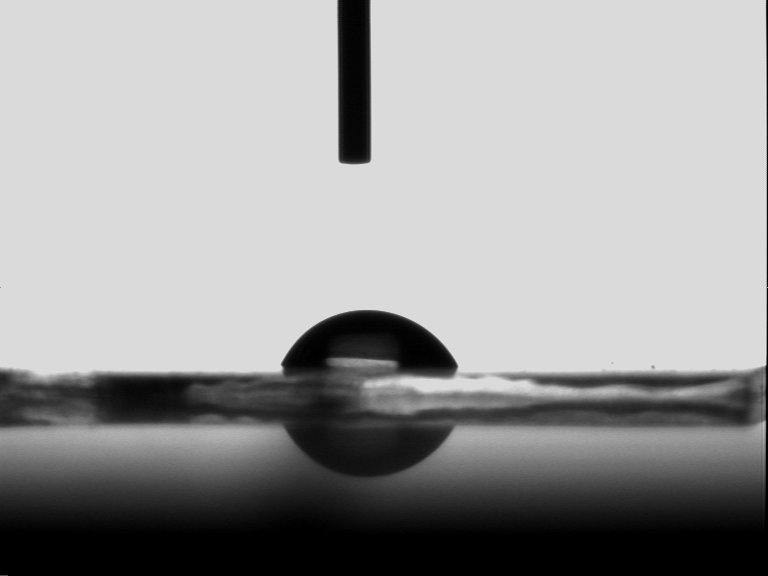

Supplement: Figure S3 — Raw data of wettability measurement using drop-shape analysis (DSA) system (DSA10Mk2, KRÜSS GmbH, Hamburg, Germany). [file peerj-07-7036-s003.zip › Fig3 Wettability/PN.bmp]

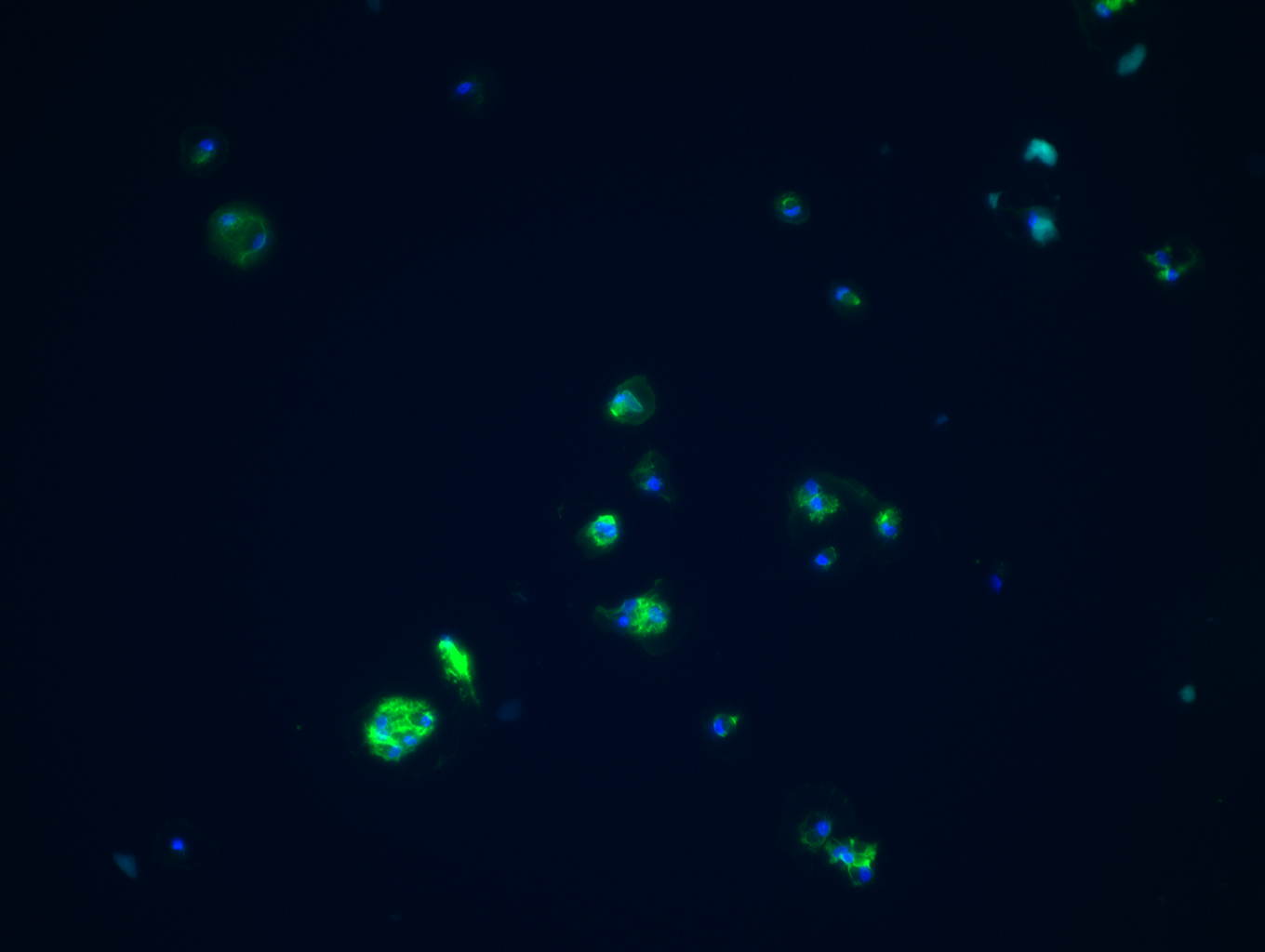

Supplement: Figure S5 — Cellular proliferation was measured using WST-8 cell counting kit (Dojindo Molecular Technologies Inc., Kumamoto, Japan). Cellular adhesion and cell morphology were examined using immunofluorescence microscopy at 1 hour after the initial seeding. DAPI (Fluoromount-G; Southern Biotech, Birmingham, Alabama, USA) was used to stain the nuclear. [file peerj-07-7036-s005.zip › Fig5 IF WST-8/FN.TIF]

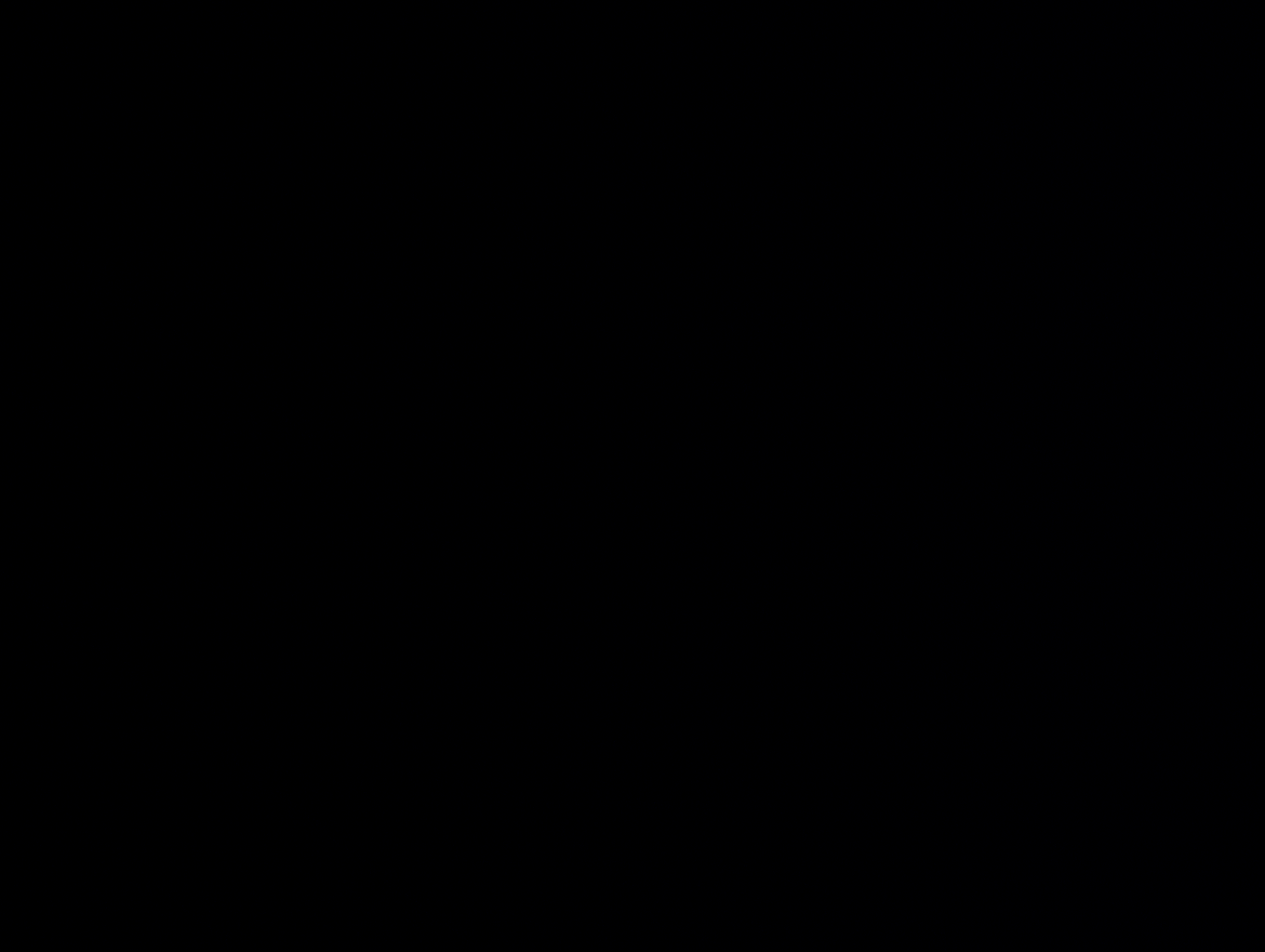

Supplement: Figure S5 — Cellular proliferation was measured using WST-8 cell counting kit (Dojindo Molecular Technologies Inc., Kumamoto, Japan). Cellular adhesion and cell morphology were examined using immunofluorescence microscopy at 1 hour after the initial seeding. DAPI (Fluoromount-G; Southern Biotech, Birmingham, Alabama, USA) was used to stain the nuclear. [file peerj-07-7036-s005.zip › Fig5 IF WST-8/NN.TIF]

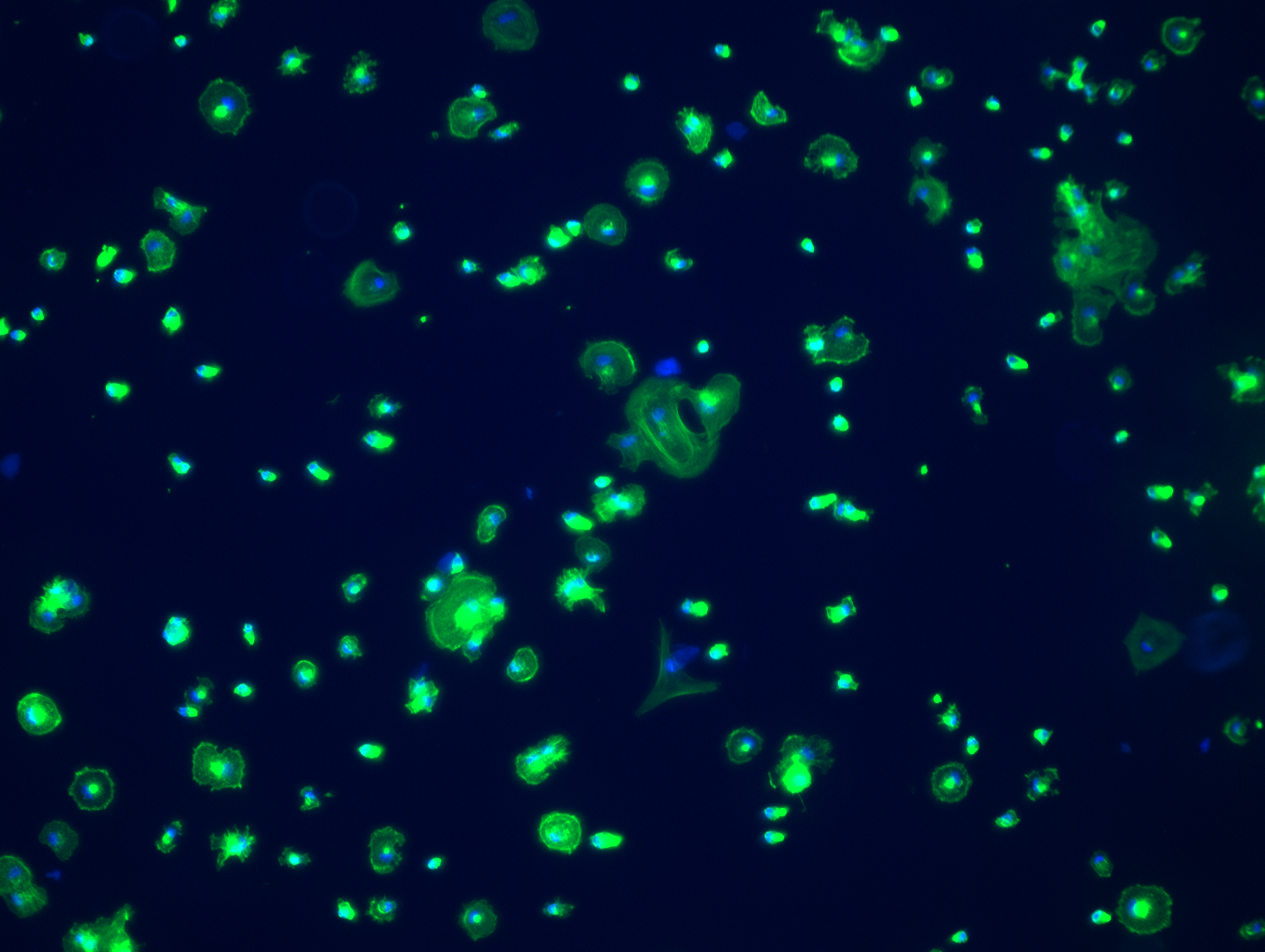

Supplement: Figure S5 — Cellular proliferation was measured using WST-8 cell counting kit (Dojindo Molecular Technologies Inc., Kumamoto, Japan). Cellular adhesion and cell morphology were examined using immunofluorescence microscopy at 1 hour after the initial seeding. DAPI (Fluoromount-G; Southern Biotech, Birmingham, Alabama, USA) was used to stain the nuclear. [file peerj-07-7036-s005.zip › Fig5 IF WST-8/PF.TIF]

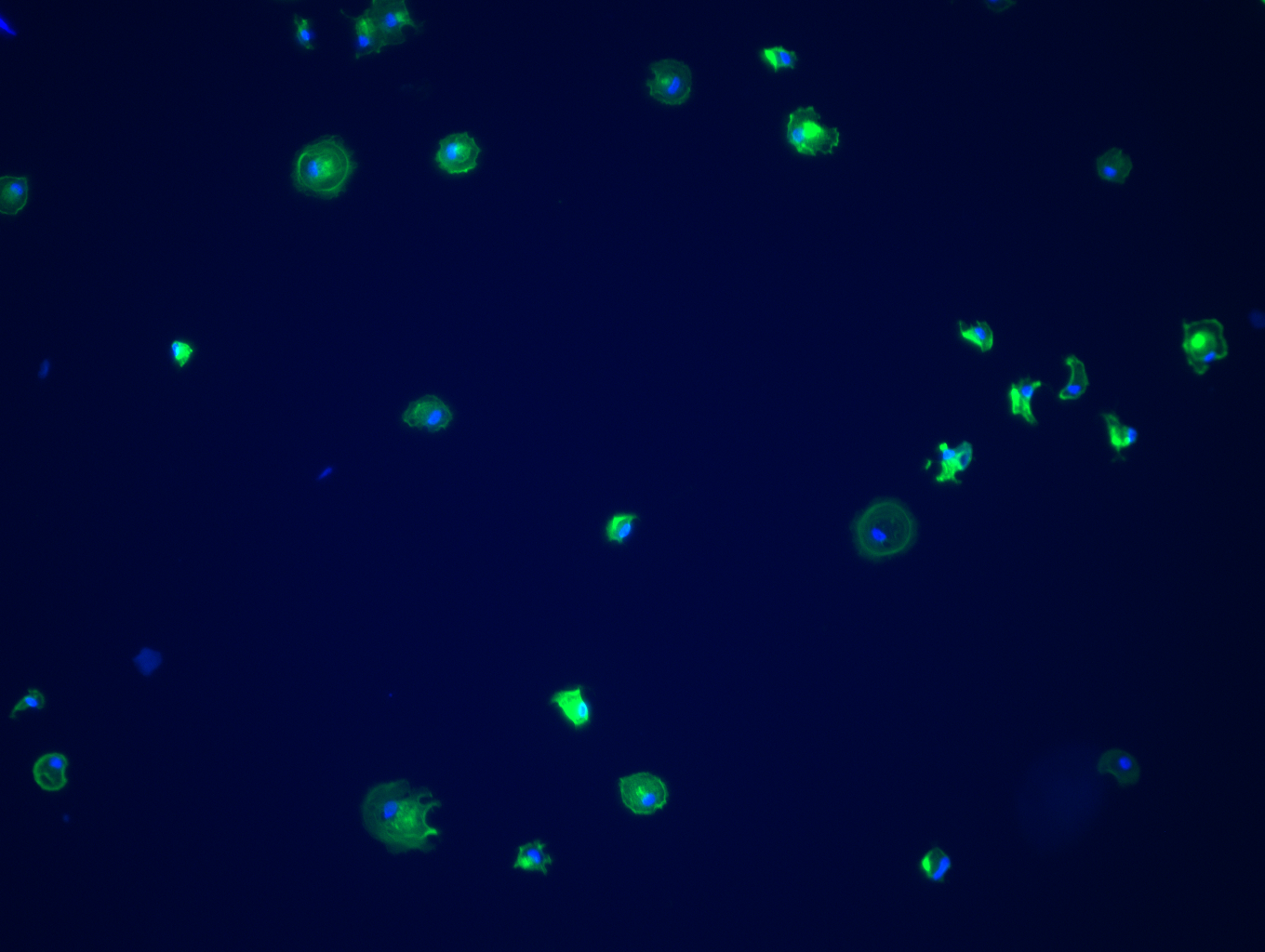

Supplement: Figure S5 — Cellular proliferation was measured using WST-8 cell counting kit (Dojindo Molecular Technologies Inc., Kumamoto, Japan). Cellular adhesion and cell morphology were examined using immunofluorescence microscopy at 1 hour after the initial seeding. DAPI (Fluoromount-G; Southern Biotech, Birmingham, Alabama, USA) was used to stain the nuclear. [file peerj-07-7036-s005.zip › Fig5 IF WST-8/PN.TIF]

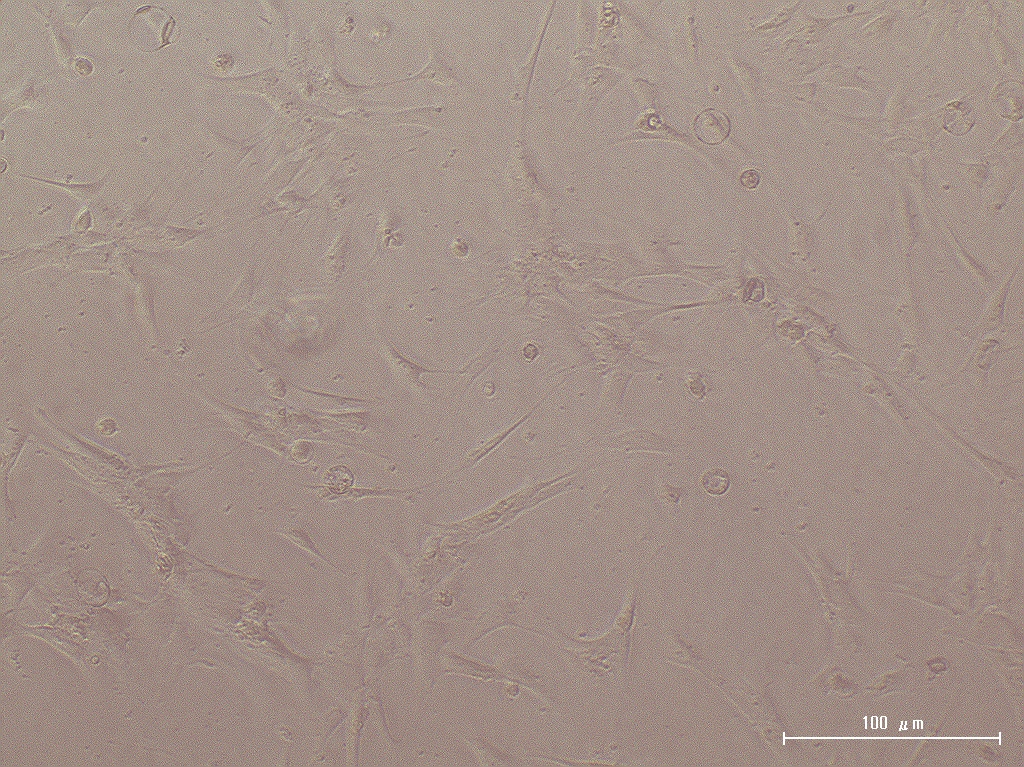

Supplement: Figure S7 — (FX380, Olympus, Tokyo, Japan). [file peerj-07-7036-s007.zip › Fig7 Polarity/PF D0.jpg]

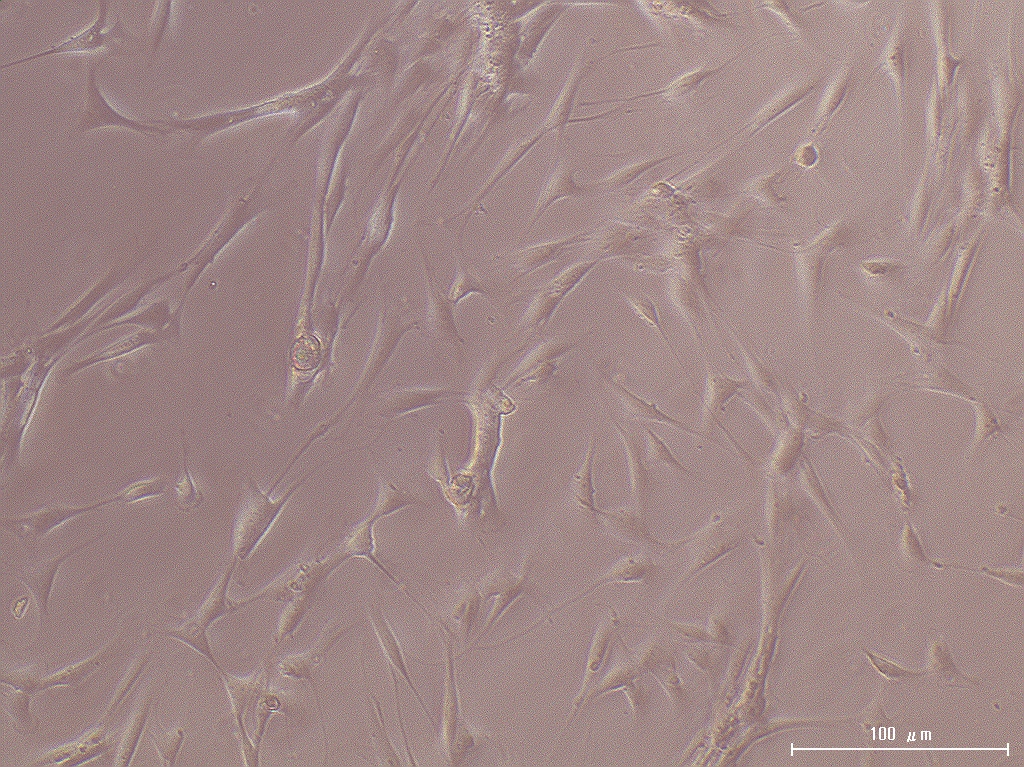

Supplement: Figure S7 — (FX380, Olympus, Tokyo, Japan). [file peerj-07-7036-s007.zip › Fig7 Polarity/PF D1.jpg]

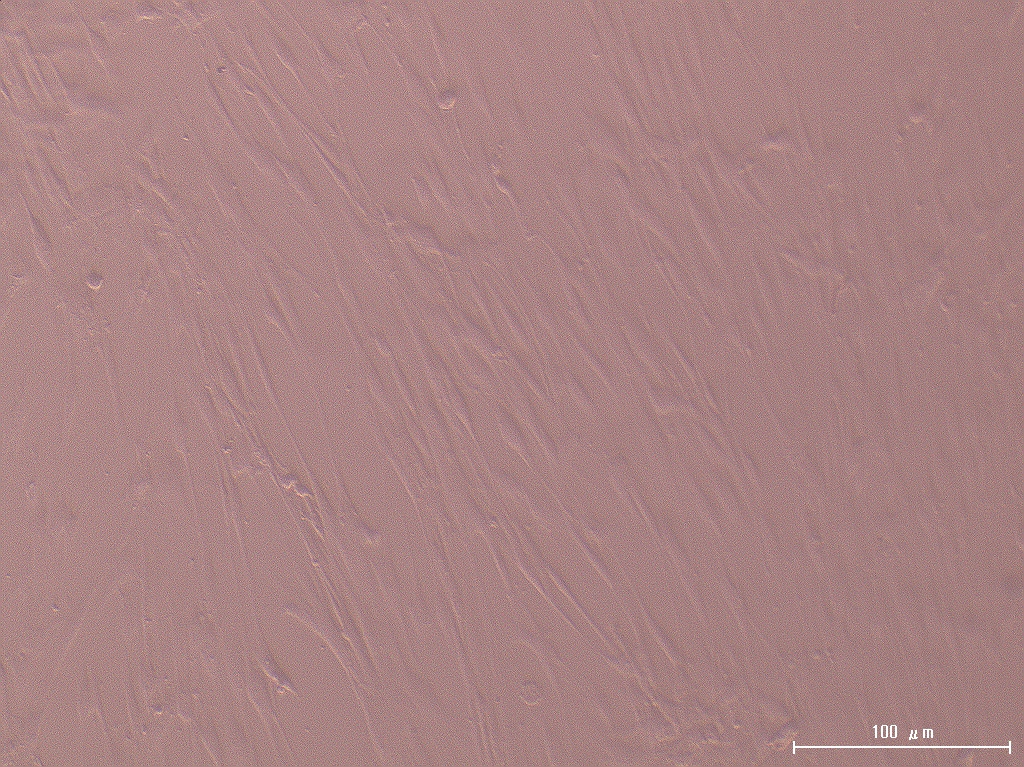

Supplement: Figure S7 — (FX380, Olympus, Tokyo, Japan). [file peerj-07-7036-s007.zip › Fig7 Polarity/PF D3.jpg]

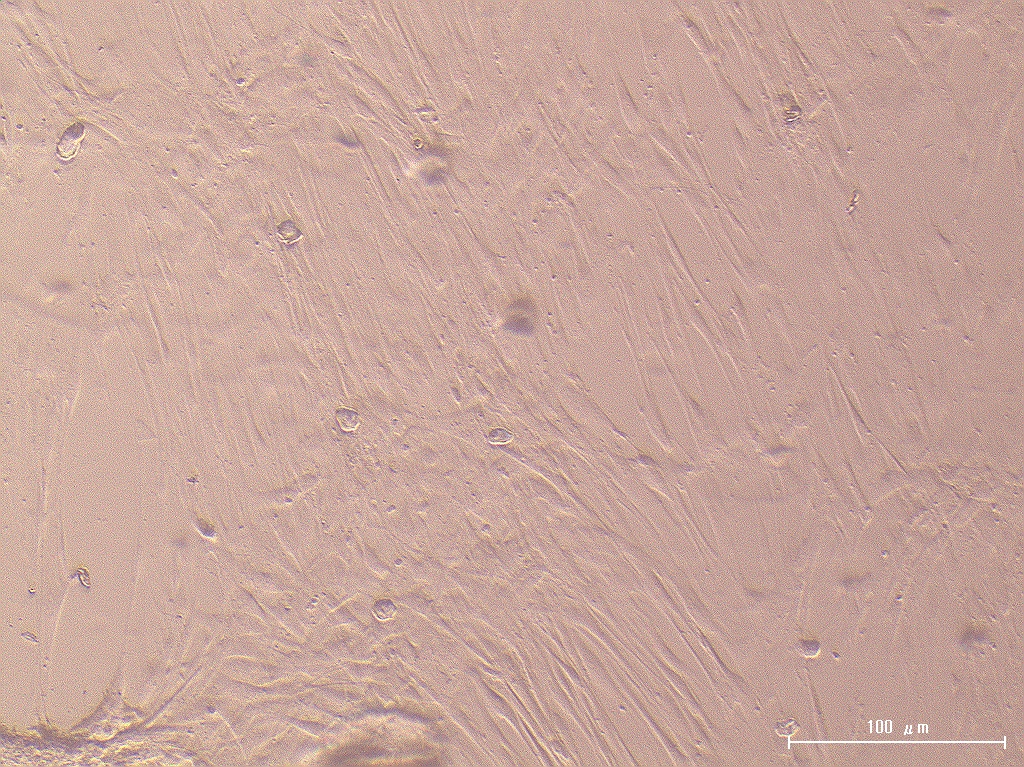

Supplement: Figure S7 — (FX380, Olympus, Tokyo, Japan). [file peerj-07-7036-s007.zip › Fig7 Polarity/PF D5.jpg]

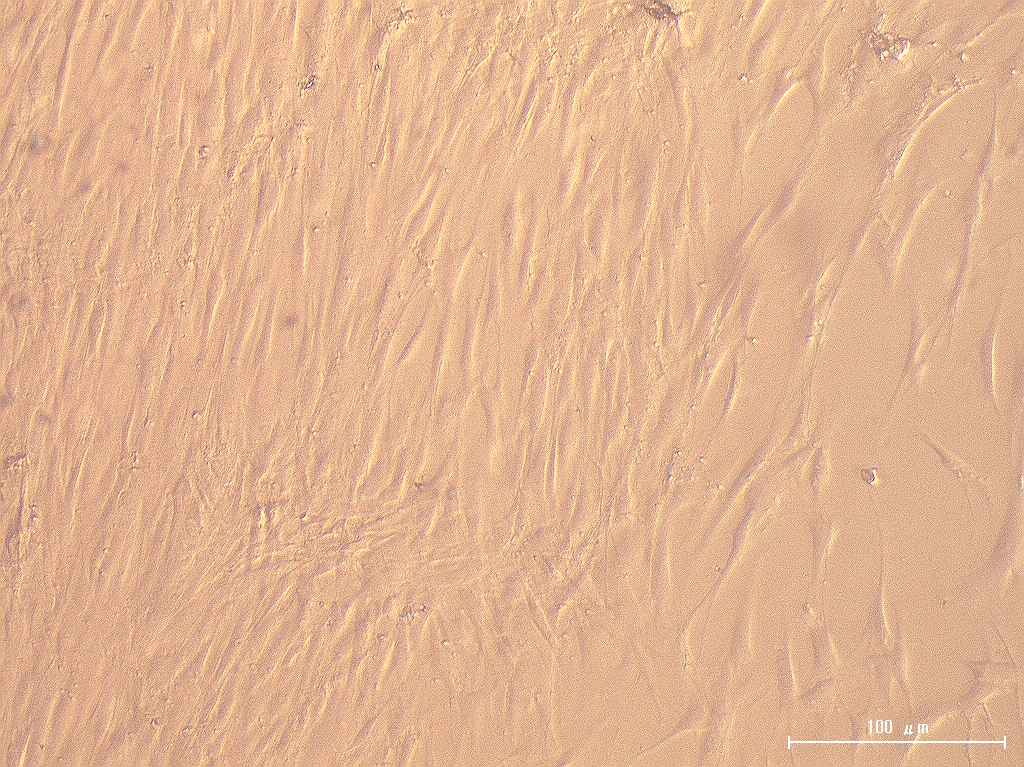

Supplement: Figure S7 — (FX380, Olympus, Tokyo, Japan). [file peerj-07-7036-s007.zip › Fig7 Polarity/PF D7.jpg]

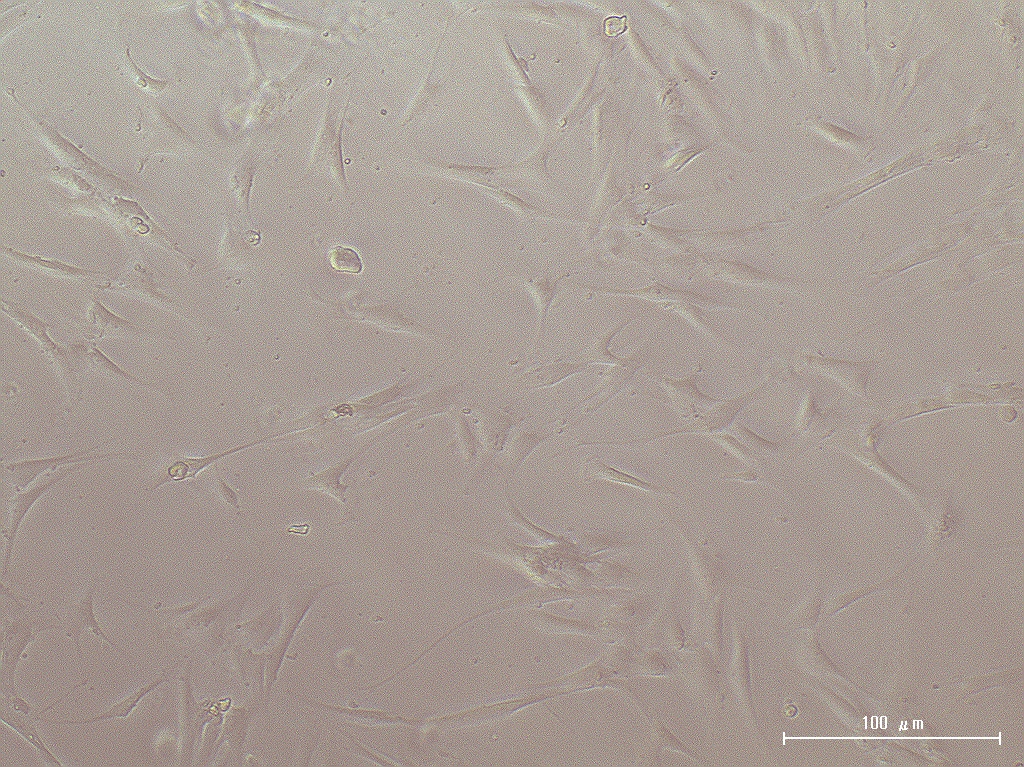

Supplement: Figure S7 — (FX380, Olympus, Tokyo, Japan). [file peerj-07-7036-s007.zip › Fig7 Polarity/PN D0.jpg]

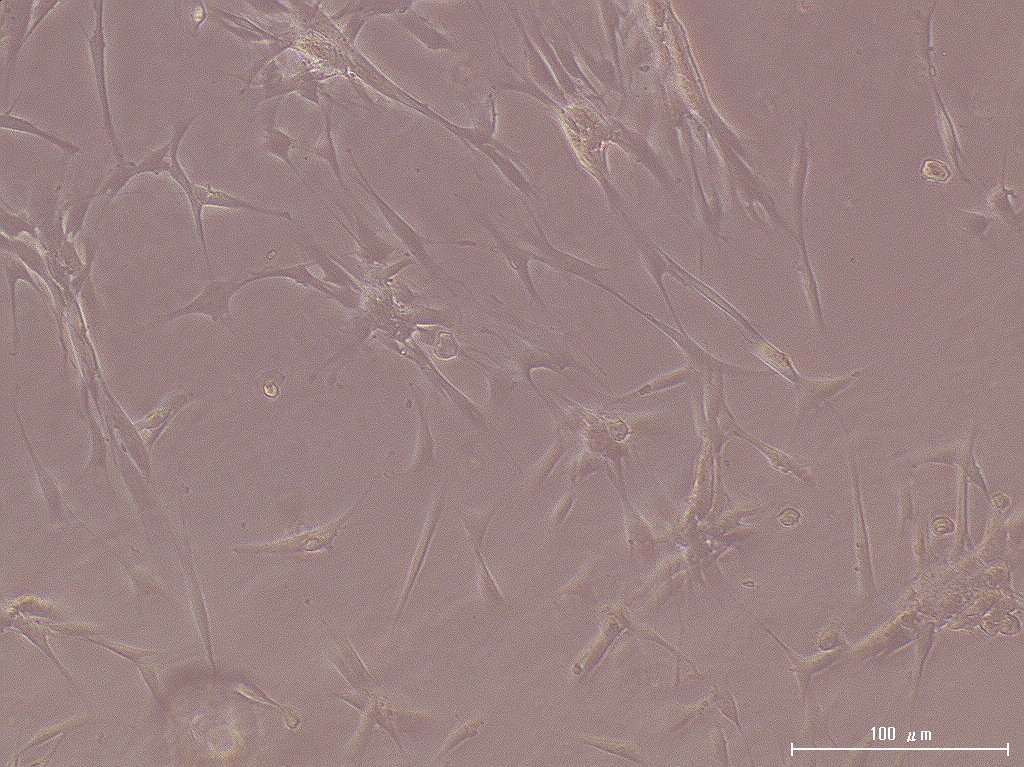

Supplement: Figure S7 — (FX380, Olympus, Tokyo, Japan). [file peerj-07-7036-s007.zip › Fig7 Polarity/PN D1.jpg]

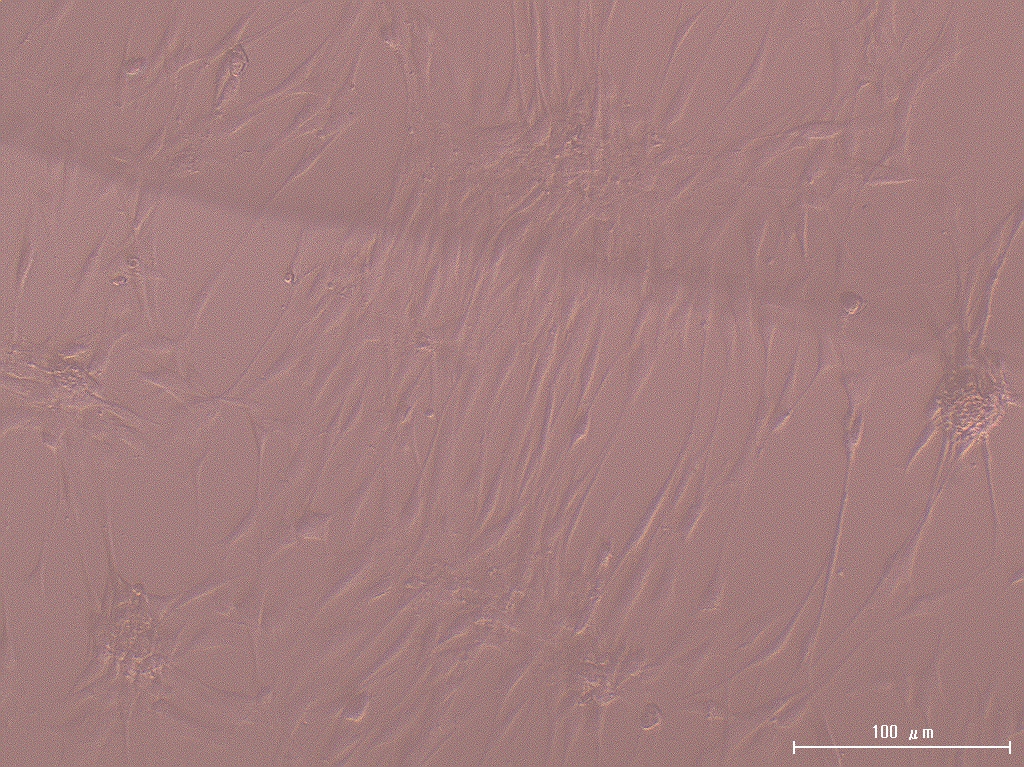

Supplement: Figure S7 — (FX380, Olympus, Tokyo, Japan). [file peerj-07-7036-s007.zip › Fig7 Polarity/PN D3.jpg]

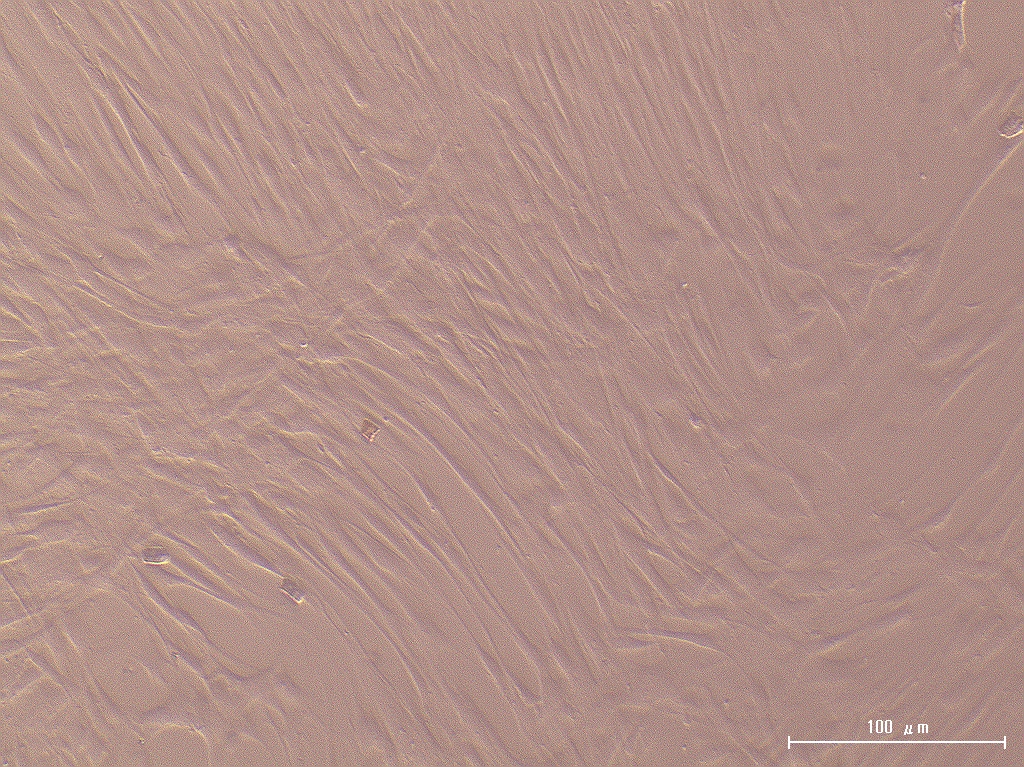

Supplement: Figure S7 — (FX380, Olympus, Tokyo, Japan). [file peerj-07-7036-s007.zip › Fig7 Polarity/PN D5.jpg]

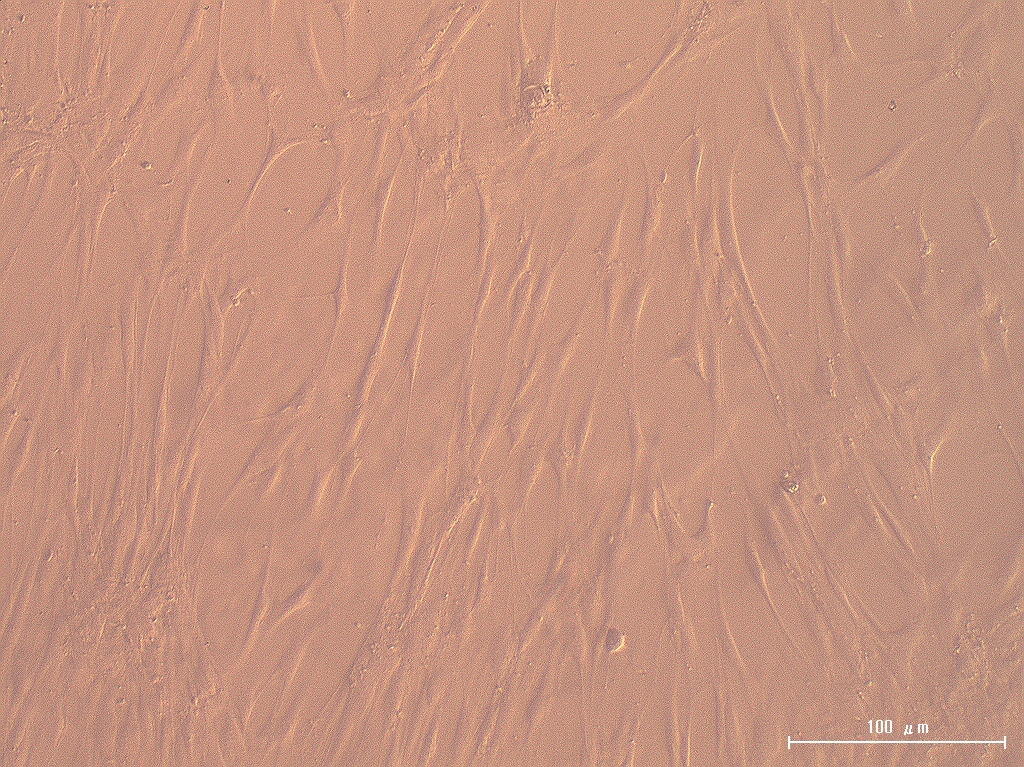

Supplement: Figure S7 — (FX380, Olympus, Tokyo, Japan). [file peerj-07-7036-s007.zip › Fig7 Polarity/PN D7.jpg]
