# Supplementary material for: Accelerated construction of an in vitro model of human periodontal ligament tissue: vacuum plasma combined with fibronectin coating and a polydimethylsiloxane matrix
Source: PeerJ. 2019 May 31;7:e7036. doi: 10.7717/peerj.7036 (PMC6546080; doi:10.7717/peerj.7036)
Supplement: Figure S4 — X-ray photoelectron spectroscopy (XPS) was performed with a PHI X-tool system (PHI X-tool, Ulvac-Phi, Kanagawa, Japan). [file peerj-07-7036-s004.zip › Fig4 ESCA/PDMS liao.pdf]

XPS Surface Analysis Result:
20140604 PDMS liao

Date:
2014/06/04

Sample Provider:

Analyst:

Instrument:
PHI X-tool

ULVAC-PHI, INC.

1. Overview

1.1 Acquisition Overview

This reporting describes the result of survey

1.2 Platen Information

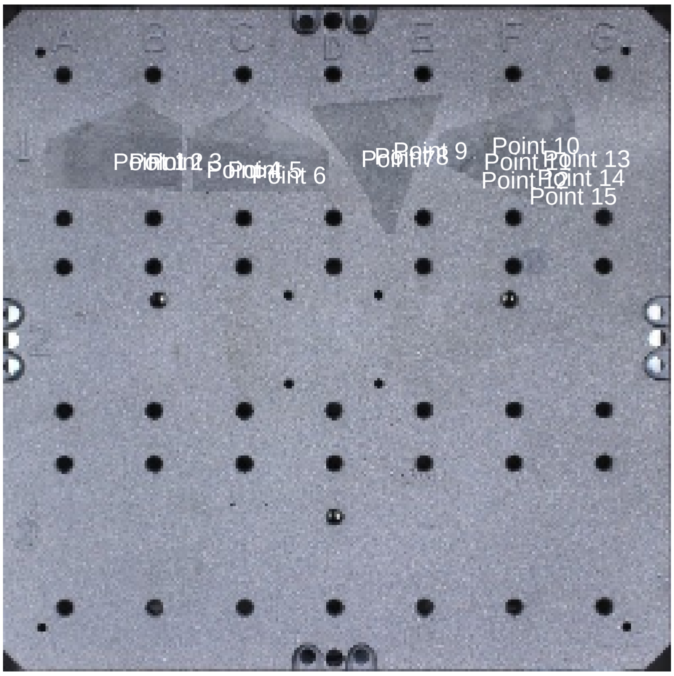

fig. 1.2.1 IntroPhoto

table. 1.2.1 Position Table

Acquisition Point	Thumbnail Phtograph	Description
Point 1	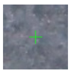	cont1
Point 2	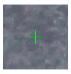	cont2
Point 3	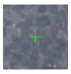	cont3
Point 4	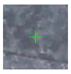	fibro1
Point 5	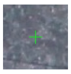	fibro2
Point 6	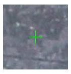	fibro3
Point 7	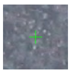	plas1
Point 8	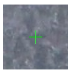	plas2
Point 9	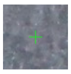	plas3

Acquisition Point	Thumbnail Phtograph	Description
Point 10	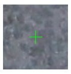	fibroplasfront1
Point 11	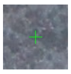	fibroplasfront2
Point 12	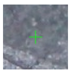	fibroplasfront3
Point 13	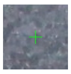	fibroplasback1
Point 14	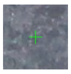	fibroplasback2
Point 15	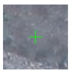	fibroplasback3

1.3 Acquisition Condition

table. 1.3.1 Survey

Acquisition Point	Point 1 2 3 4 5 6 7 8 9 10 11 12 13 14 15
Instrument	PHI X-tool
Xray Source	Al Kα
Xray Condition	15kV 25W
Acquisition Area	108 μm
Take-Off Angle	45 deg.
Neutralization Condition	1.2eV 20.0μA
Clean Sputterred	Not Sputter Cleaned

table. 1.3.2 Region

Name	Range(eV)	PassEnergy(eV)	StepSize(eV)	Time(s)
SURVEY	1300.00 - 0.00eV	224.00eV	0.800eV	20.000s

table. 1.3.3 Narrow

Acquisition Point	Point 1 2 3 4 5 6 7 8<br>9 10 11 12 13 14 15
Instrument	PHI X-tool
Xray Source	Al Kα
Xray Condition	15kV 5W
Acquisition Area	19 μm
Take-Off Angle	45 deg.
Neutralization Condition	1.2eV 20.0μA
Clean Sputterred	Not Sputter Cleaned

table. 1.3.4 Region

Name	Range(eV)	PassEnergy(eV)	StepSize(eV)	Time(s)
H1s	26.00 - 6.00eV	55.00eV	0.200eV	20.000s
C1s	298.00 - 278.00eV	280.00eV	1.000eV	20.000s
N1s	411.00 - 391.00eV	280.00eV	1.000eV	20.000s
O1s	543.00 - 523.00eV	280.00eV	1.000eV	20.000s
Si2p	114.00 - 94.00eV	280.00eV	1.000eV	20.000s

2. Result

2.1 Atomic Concentartion Table

2.1.1 Survey

Acquisition Point:1
Area Comment:cont1
File Path:20140604_140102¥20140604 PDMS liao¥14604_142521.spe
Acquisition Point:2
Area Comment:cont2
File Path:20140604_140102¥20140604 PDMS liao¥14604_143038.spe
Acquisition Point:3
Area Comment:cont3
File Path:20140604_140102¥20140604 PDMS liao¥14604_143315.spe
Acquisition Point:4
Area Comment:fibro1
File Path:20140604_140102¥20140604 PDMS liao¥14604_143707.spe
Acquisition Point:5
Area Comment:fibro2
File Path:20140604_140102¥20140604 PDMS liao¥14604_144111.spe
Acquisition Point:6
Area Comment:fibro3
File Path:20140604_140102¥20140604 PDMS liao¥14604_144505.spe
Acquisition Point:7
Area Comment:plas1
File Path:20140604_140102¥20140604 PDMS liao¥14604_144853.spe
Acquisition Point:8
Area Comment:plas2
File Path:20140604_140102¥20140604 PDMS liao¥14604_145258.spe
Acquisition Point:9
Area Comment:plas3
File Path:20140604_140102¥20140604 PDMS liao¥14604_145529.spe
Acquisition Point:10
Area Comment:fibroplasfront1
File Path:20140604_140102¥20140604 PDMS liao¥14604_145835.spe
Acquisition Point:11
Area Comment:fibroplasfront2
File Path:20140604_140102¥20140604 PDMS liao¥14604_150235.spe
Acquisition Point:12
Area Comment:fibroplasfront3
File Path:20140604_140102¥20140604 PDMS liao¥14604_150511.spe
Acquisition Point:13
Area Comment:fibroplasback1
File Path:20140604_140102¥20140604 PDMS liao¥14604_150859.spe
Acquisition Point:14
Area Comment:fibroplasback2
File Path:20140604_140102¥20140604 PDMS liao¥14604_151256.spe
Acquisition Point:15
Area Comment:fibroplasback3
File Path:20140604_140102¥20140604 PDMS liao¥14604_151649.spe

Acquisition Point	C1s(%)	O1s(%)	Si2p(%)	S2p(%)	N1s(%)
1	30.3	45.5	24.2	0.0	-
2	31.7	43.4	24.9	-	-
3	38.3	37.3	24.3	-	-
4	40.4	36.8	22.9	-	-

Acquisition Point	C1s(%)	O1s(%)	Si2p(%)	S2p(%)	N1s(%)
5	45.2	32.5	19.2	-	3.1
6	47.2	29.6	22.2	-	1.0
7	10.6	63.7	25.8	-	-
8	14.6	60.7	24.7	-	-
9	15.2	59.4	25.4	-	-
10	34.5	40.4	25.1	-	-
11	36.8	38.0	25.2	-	-
12	33.3	41.5	25.3	-	-
13	34.7	39.2	26.0	-	-
14	46.5	32.7	20.8	-	-
15	38.4	37.0	24.6	-	-

2.1.2 Narrow

Acquisition Point:1
Area Comment:cont1
File Path:20140604_140102¥20140604 PDMS liao¥14604_142948.spe
Acquisition Point:2
Area Comment:cont2
File Path:20140604_140102¥20140604 PDMS liao¥14604_143237.spe
Acquisition Point:3
Area Comment:cont3
File Path:20140604_140102¥20140604 PDMS liao¥14604_143622.spe
Acquisition Point:4
Area Comment:fibro1
File Path:20140604_140102¥20140604 PDMS liao¥14604_144030.spe
Acquisition Point:5
Area Comment:fibro2
File Path:20140604_140102¥20140604 PDMS liao¥14604_144427.spe
Acquisition Point:6
Area Comment:fibro3
File Path:20140604_140102¥20140604 PDMS liao¥14604_144812.spe
Acquisition Point:7
Area Comment:plas1
File Path:20140604_140102¥20140604 PDMS liao¥14604_145213.spe
Acquisition Point:8
Area Comment:plas2
File Path:20140604_140102¥20140604 PDMS liao¥14604_145449.spe
Acquisition Point:9
Area Comment:plas3
File Path:20140604_140102¥20140604 PDMS liao¥14604_145757.spe
Acquisition Point:10
Area Comment:fibroplasfront1
File Path:20140604_140102¥20140604 PDMS liao¥14604_150156.spe
Acquisition Point:11
Area Comment:fibroplasfront2
File Path:20140604_140102¥20140604 PDMS liao¥14604_150433.spe
Acquisition Point:12
Area Comment:fibroplasfront3
File Path:20140604_140102¥20140604 PDMS liao¥14604_150820.spe
Acquisition Point:13
Area Comment:fibroplasback1
File Path:20140604_140102¥20140604 PDMS liao¥14604_151217.spe
Acquisition Point:14
Area Comment:fibroplasback2
File Path:20140604_140102¥20140604 PDMS liao¥14604_151610.spe
Acquisition Point:15
Area Comment:fibroplasback3
File Path:20140604_140102¥20140604 PDMS liao¥14604_151956.spe

Acquisition Point	H1s(%)	C1s(%)	N1s(%)	O1s(%)	Si2p(%)
1	0.0	28.1	0.0	46.3	25.7
2	99.6	0.1	0.0	0.2	0.1
3	0.0	33.5	0.7	39.5	26.2
4	99.1	0.4	0.0	0.3	0.2
5	0.0	52.0	0.0	27.7	20.3
6	0.0	38.8	7.2	32.0	21.9
7	97.4	0.2	0.0	1.7	0.7
8	94.5	0.6	0.0	3.5	1.4

Acquisition Point	H1s(%)	C1s(%)	N1s(%)	O1s(%)	Si2p(%)
9	0.0	12.4	0.0	64.3	23.3
10	0.0	34.8	2.2	37.5	25.5
11	99.4	0.2	0.0	0.2	0.2
12	99.6	0.1	0.0	0.2	0.1
13	99.5	0.2	0.0	0.2	0.1
14	99.8	0.1	0.0	0.1	0.0
15	0.0	36.3	0.9	38.0	24.7

2.2 Survey

table. 2.2.1 File Table

Position	File Path
1	20140604_140102¥20140604 PDMS liao¥14604_142521.spe
2	20140604_140102¥20140604 PDMS liao¥14604_143038.spe
3	20140604_140102¥20140604 PDMS liao¥14604_143315.spe
4	20140604_140102¥20140604 PDMS liao¥14604_143707.spe
5	20140604_140102¥20140604 PDMS liao¥14604_144111.spe
6	20140604_140102¥20140604 PDMS liao¥14604_144505.spe
7	20140604_140102¥20140604 PDMS liao¥14604_144853.spe
8	20140604_140102¥20140604 PDMS liao¥14604_145258.spe
9	20140604_140102¥20140604 PDMS liao¥14604_145529.spe
10	20140604_140102¥20140604 PDMS liao¥14604_145835.spe
11	20140604_140102¥20140604 PDMS liao¥14604_150235.spe
12	20140604_140102¥20140604 PDMS liao¥14604_150511.spe
13	20140604_140102¥20140604 PDMS liao¥14604_150859.spe
14	20140604_140102¥20140604 PDMS liao¥14604_151256.spe
15	20140604_140102¥20140604 PDMS liao¥14604_151649.spe

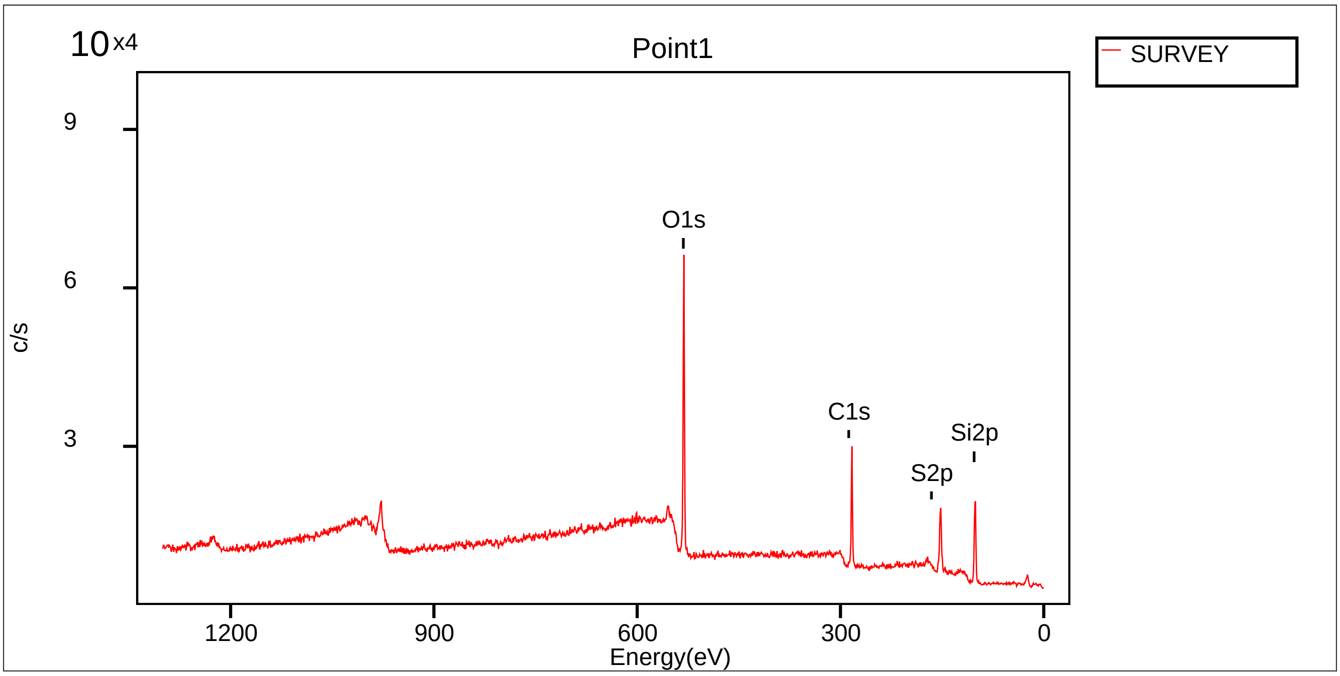

fig. 2.2.1 Survey Point1

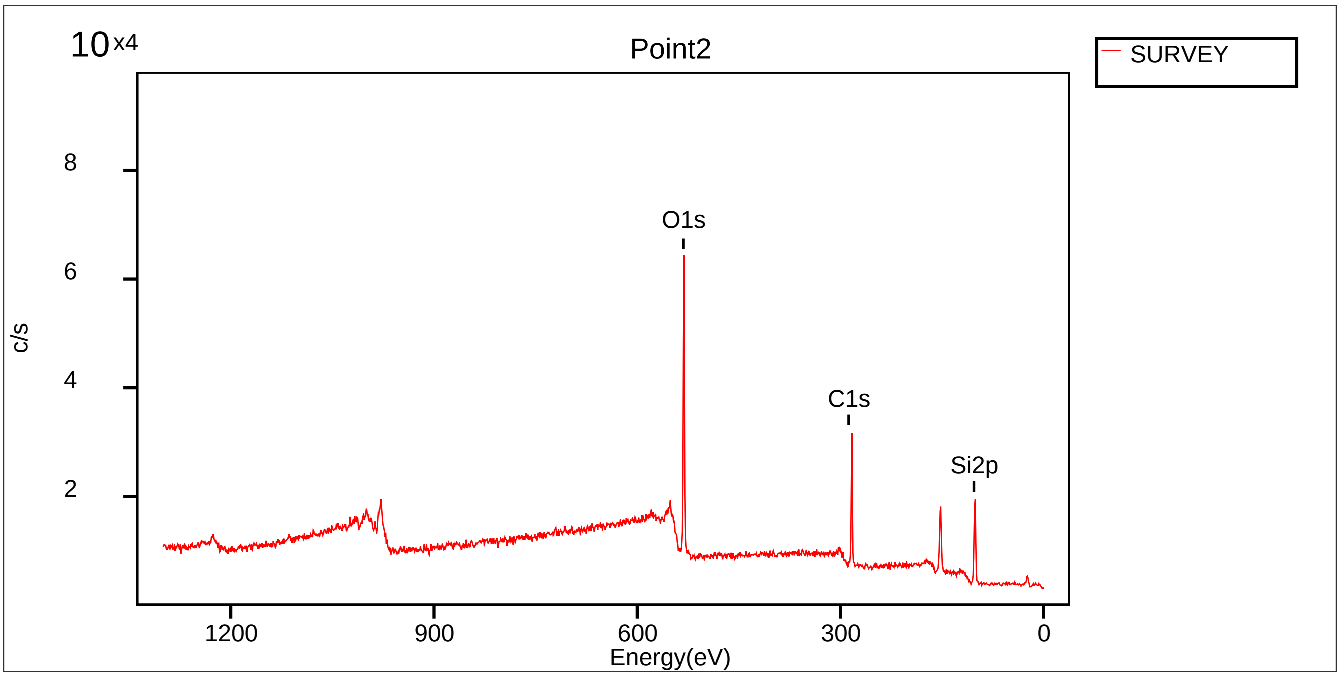

fig. 2.2.2 Survey Point2

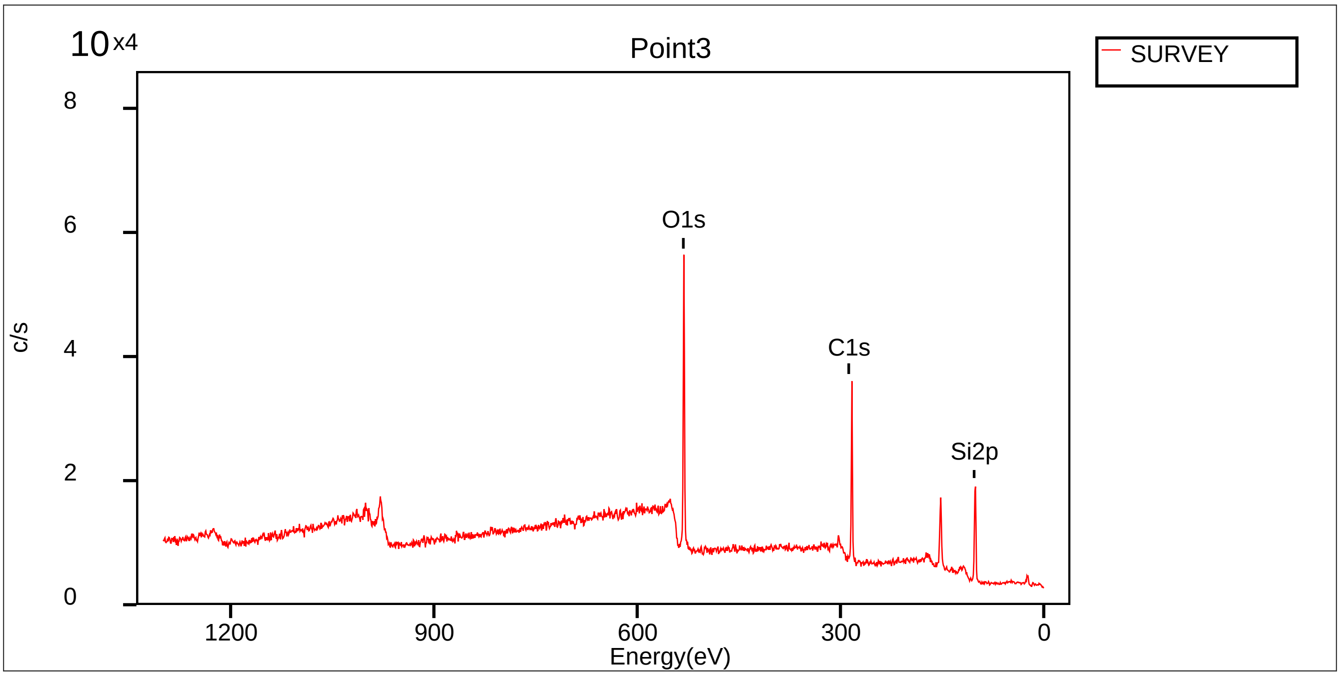

fig. 2.2.3 Survey Point3

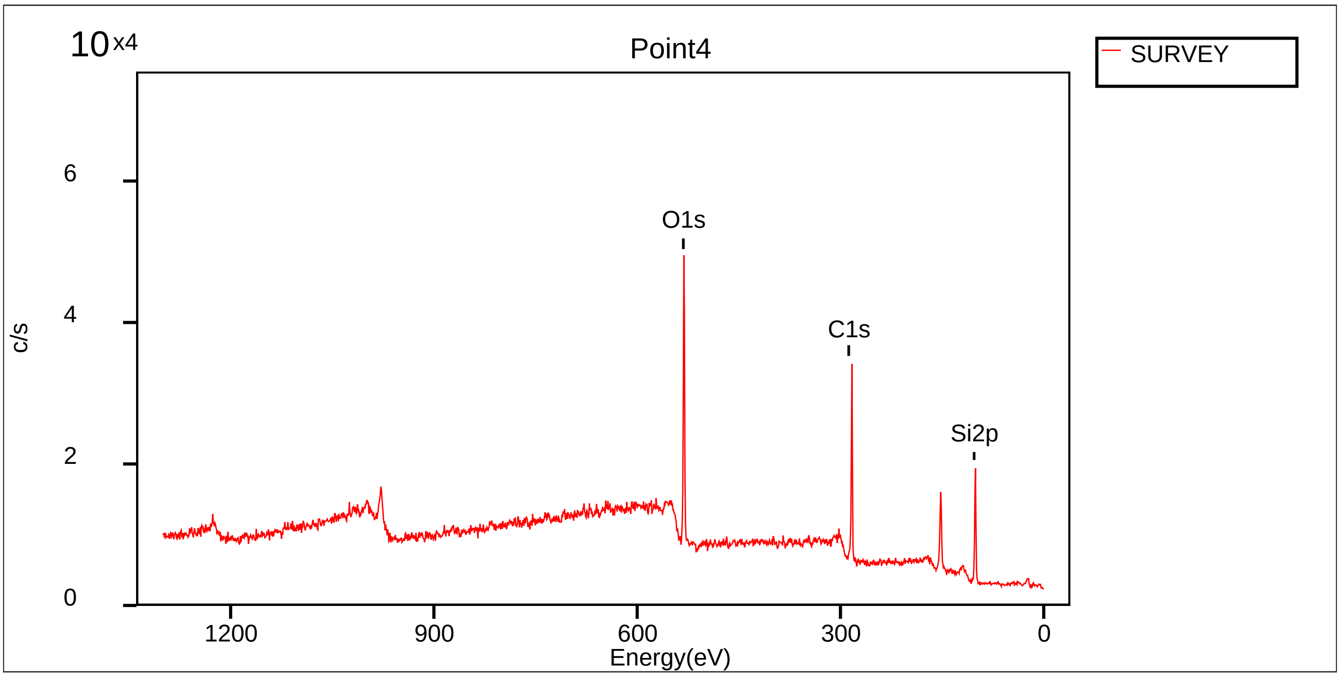

fig. 2.2.4 Survey Point4

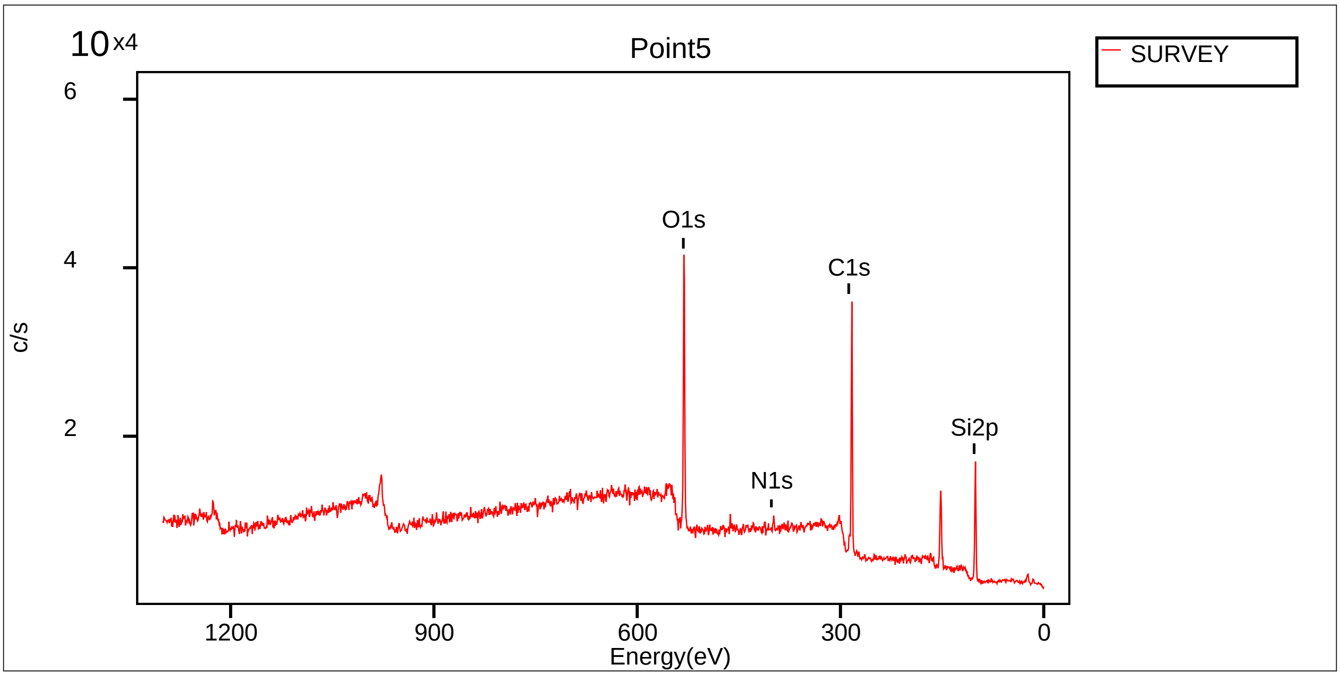

fig. 2.2.5 Survey Point5

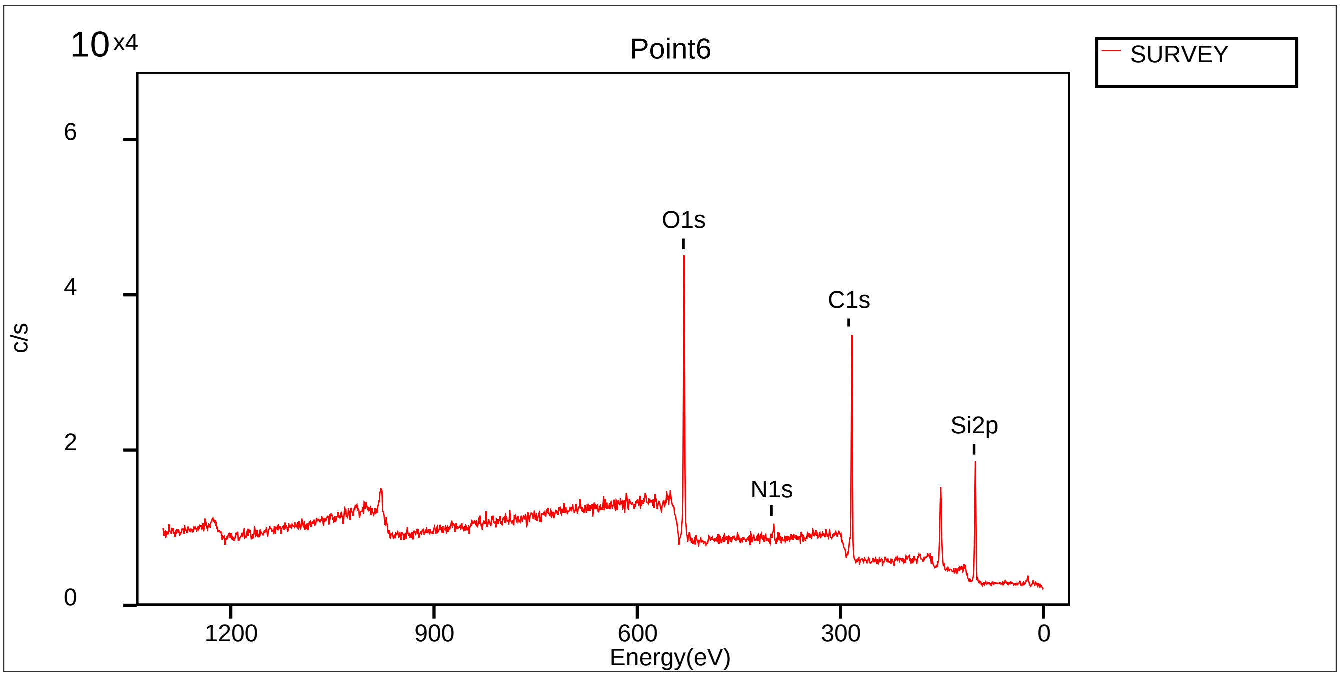

fig. 2.2.6 Survey Point6

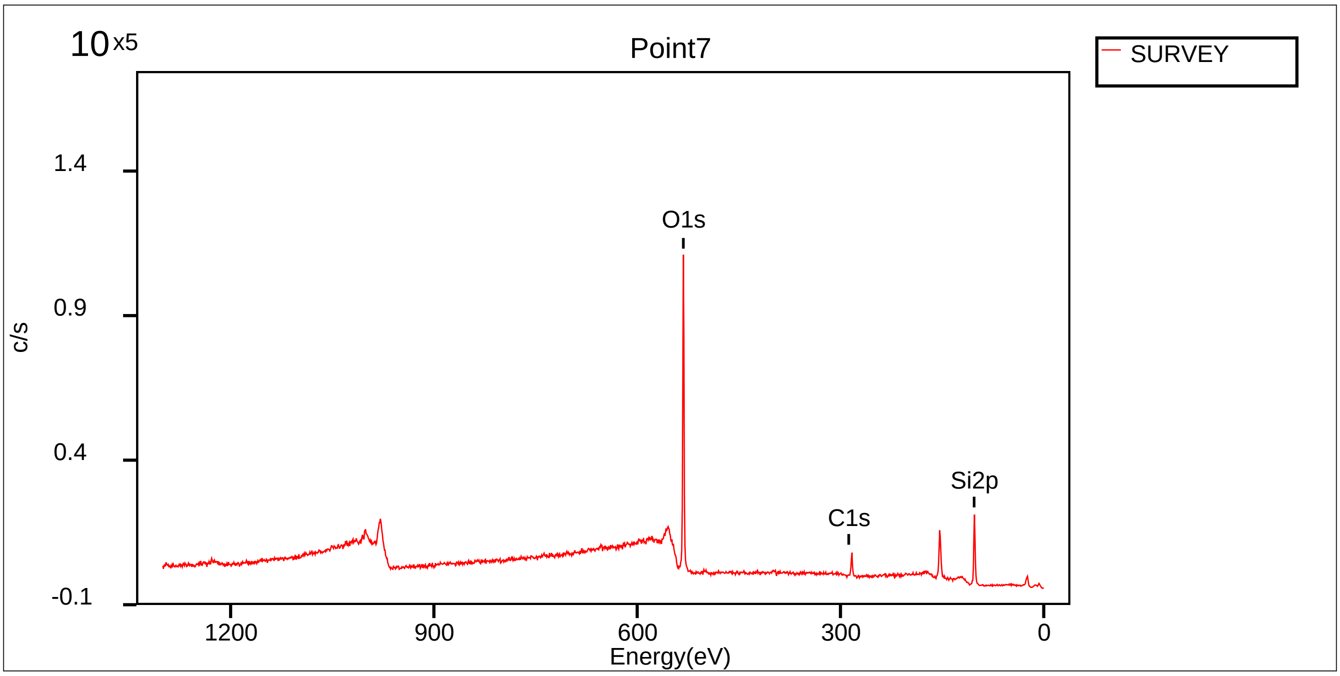

fig. 2.2.7 Survey Point7

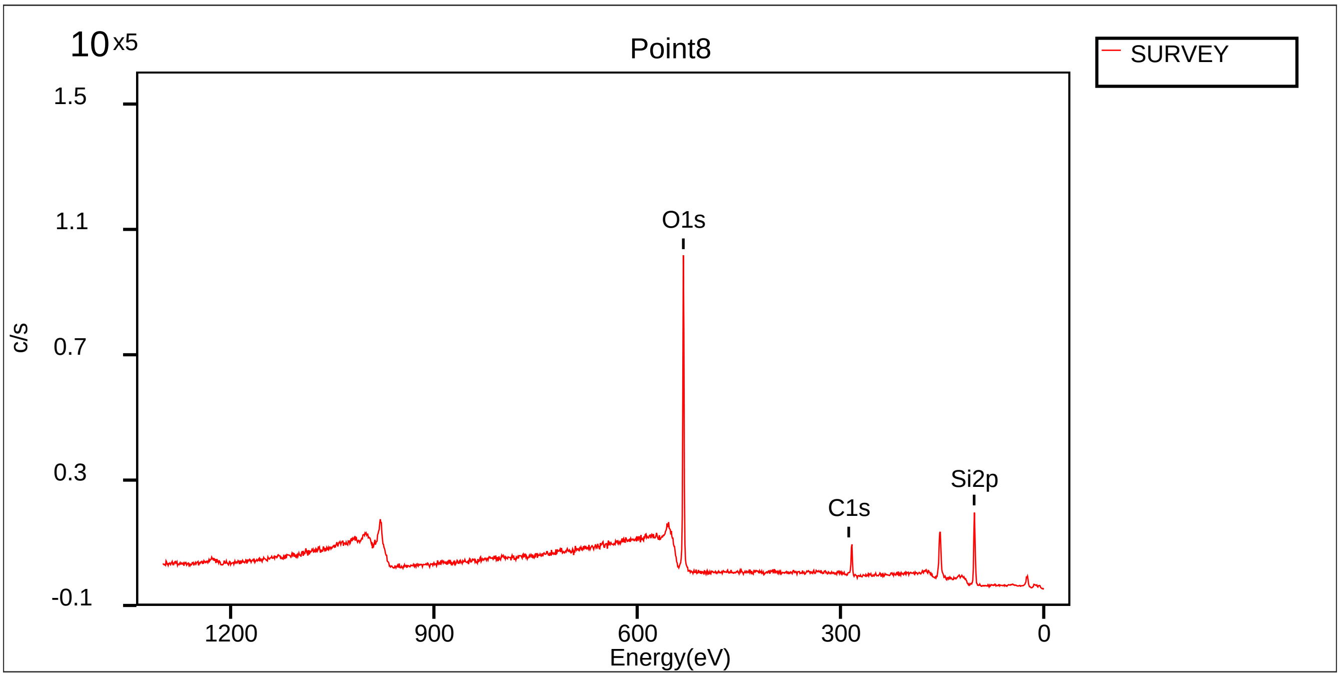

fig. 2.2.8 Survey Point8

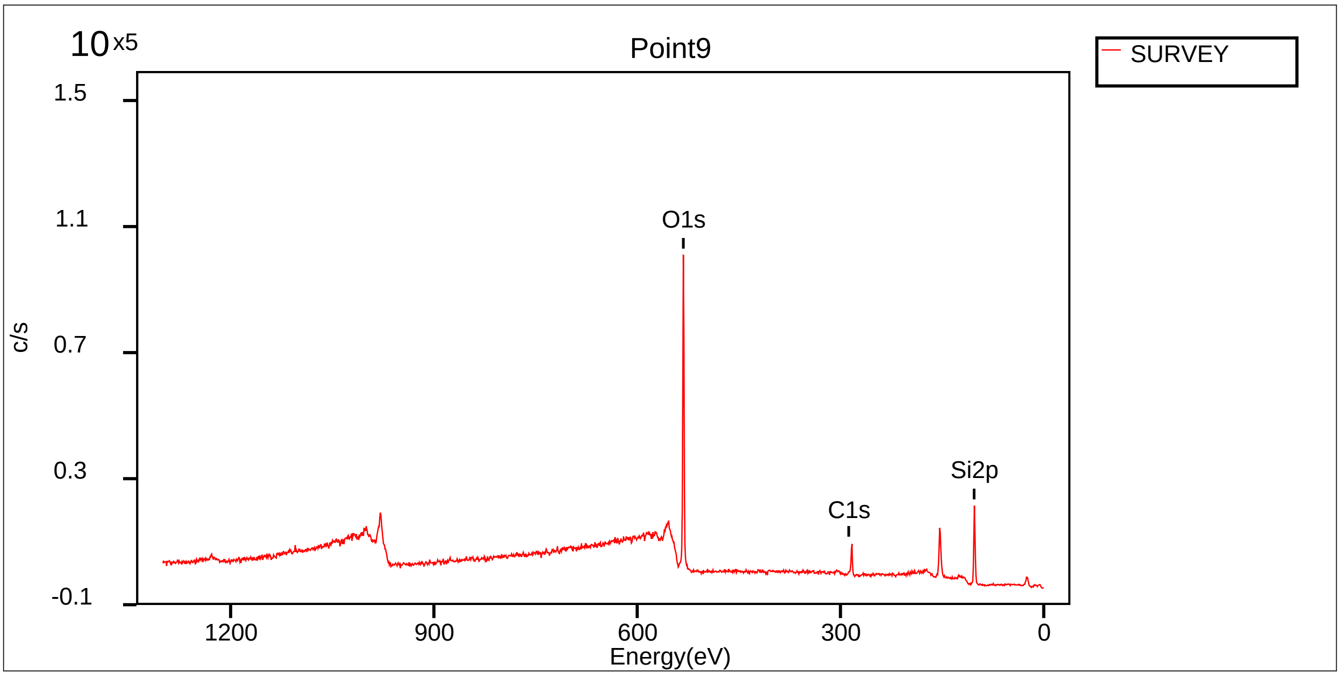

fig. 2.2.9 Survey Point9

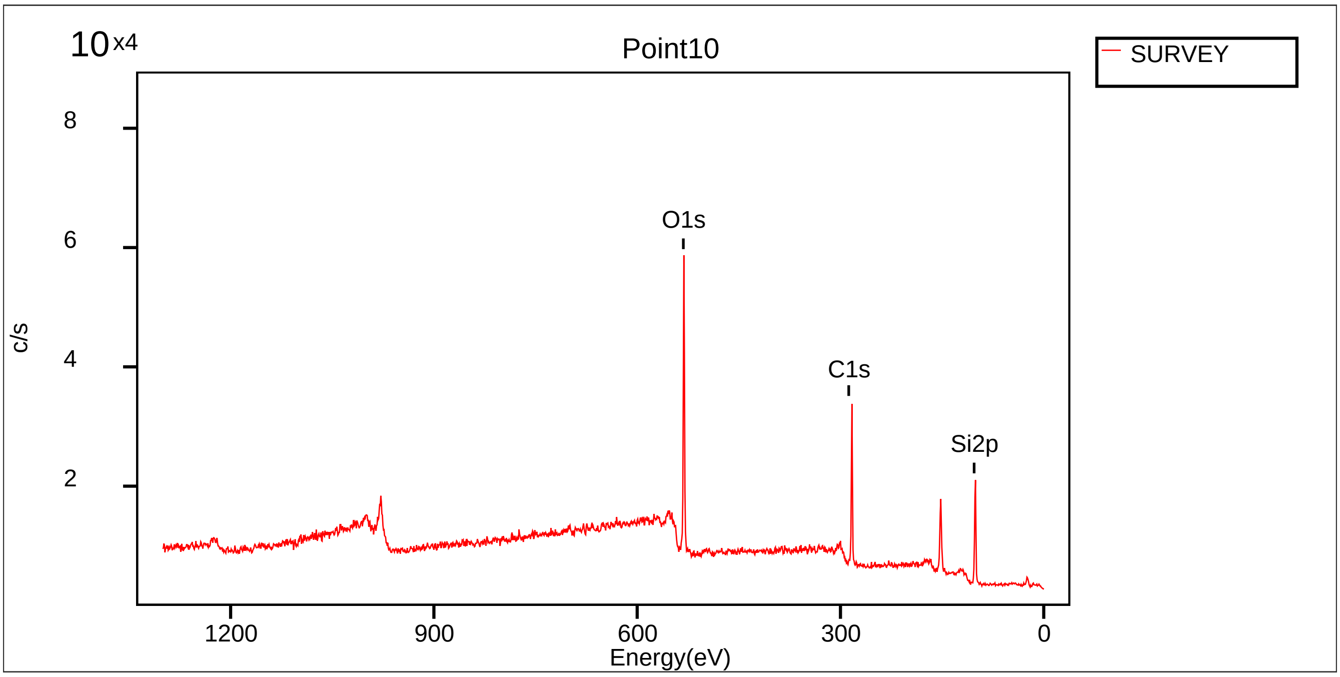

fig. 2.2.10 Survey Point10

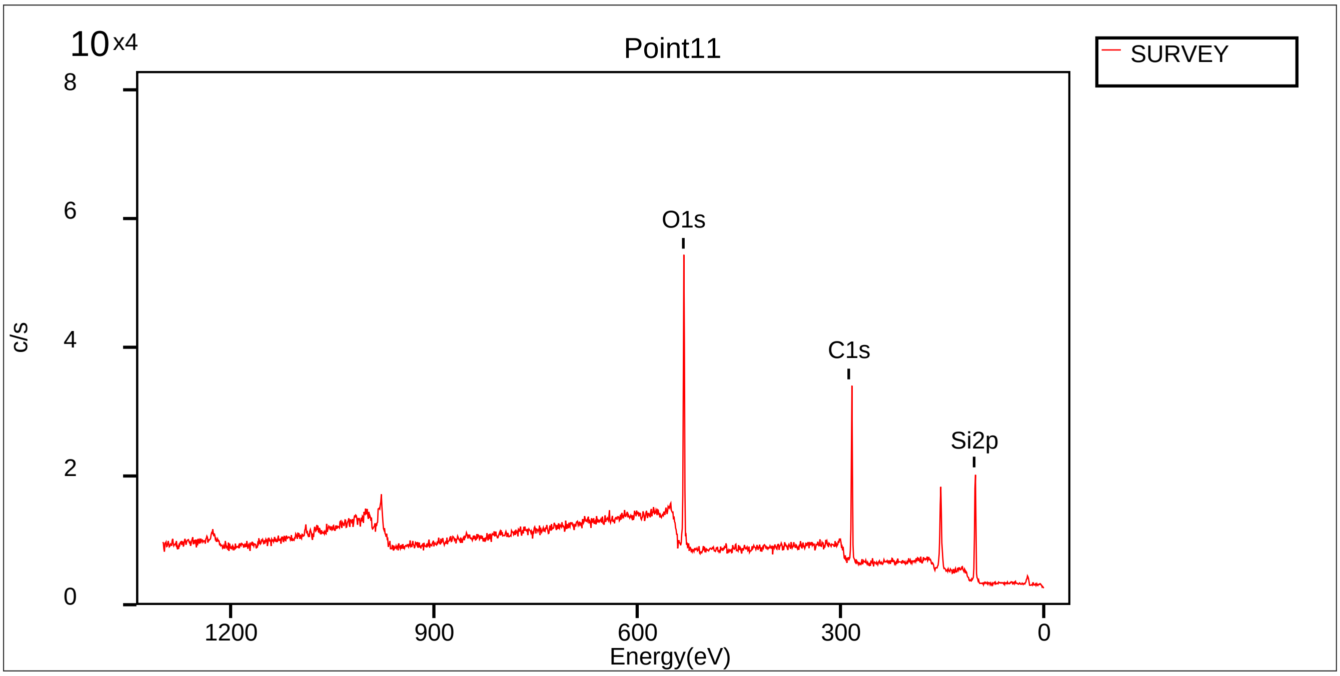

fig. 2.2.11 Survey Point11

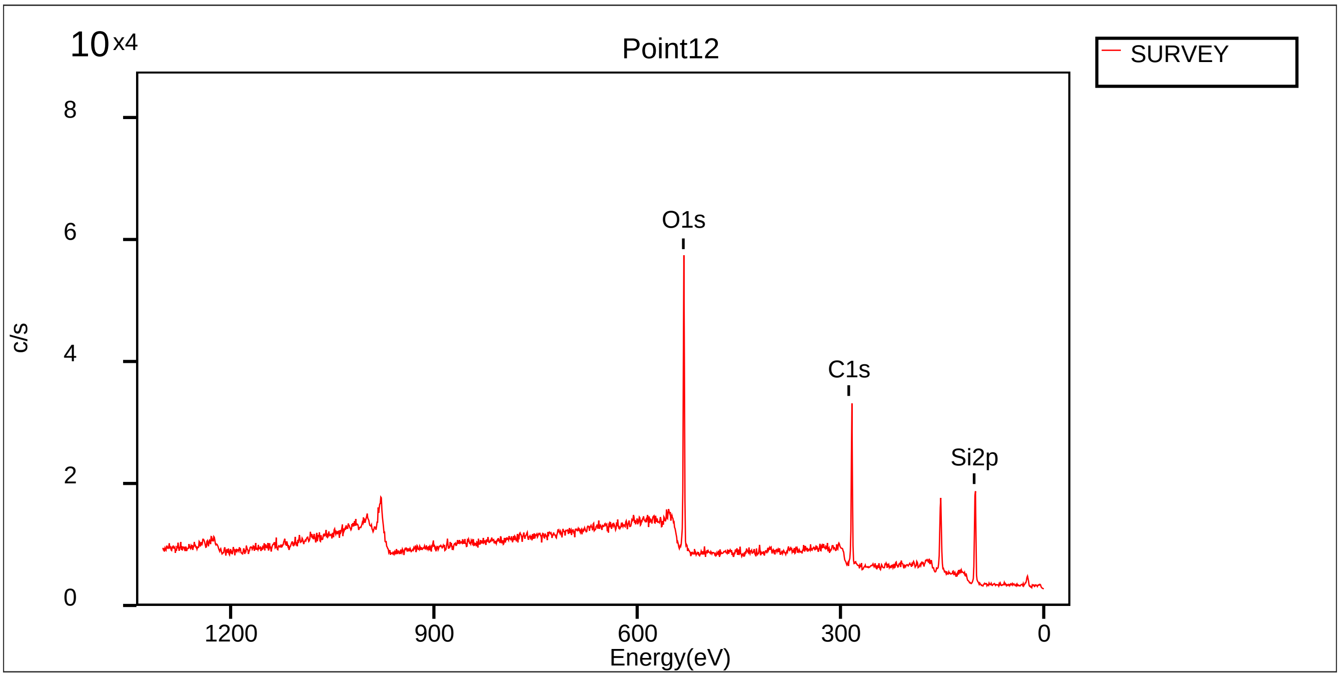

fig. 2.2.12 Survey Point12

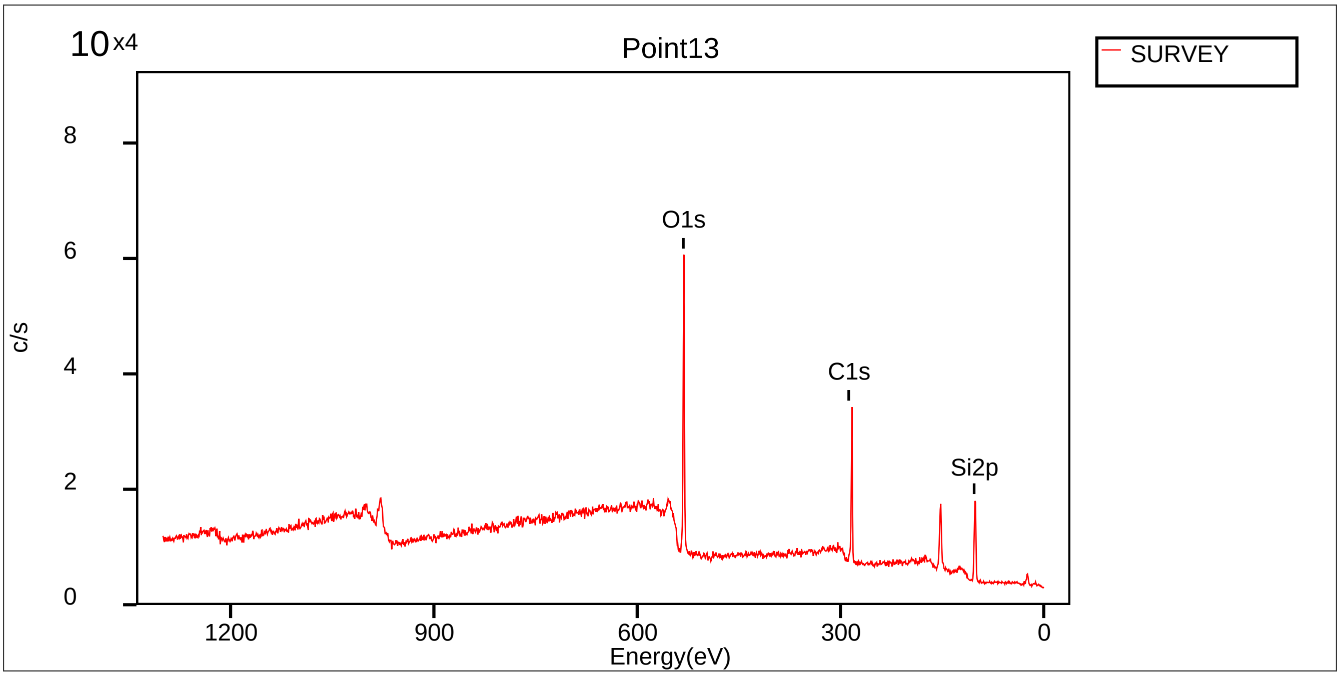

fig. 2.2.13 Survey Point13

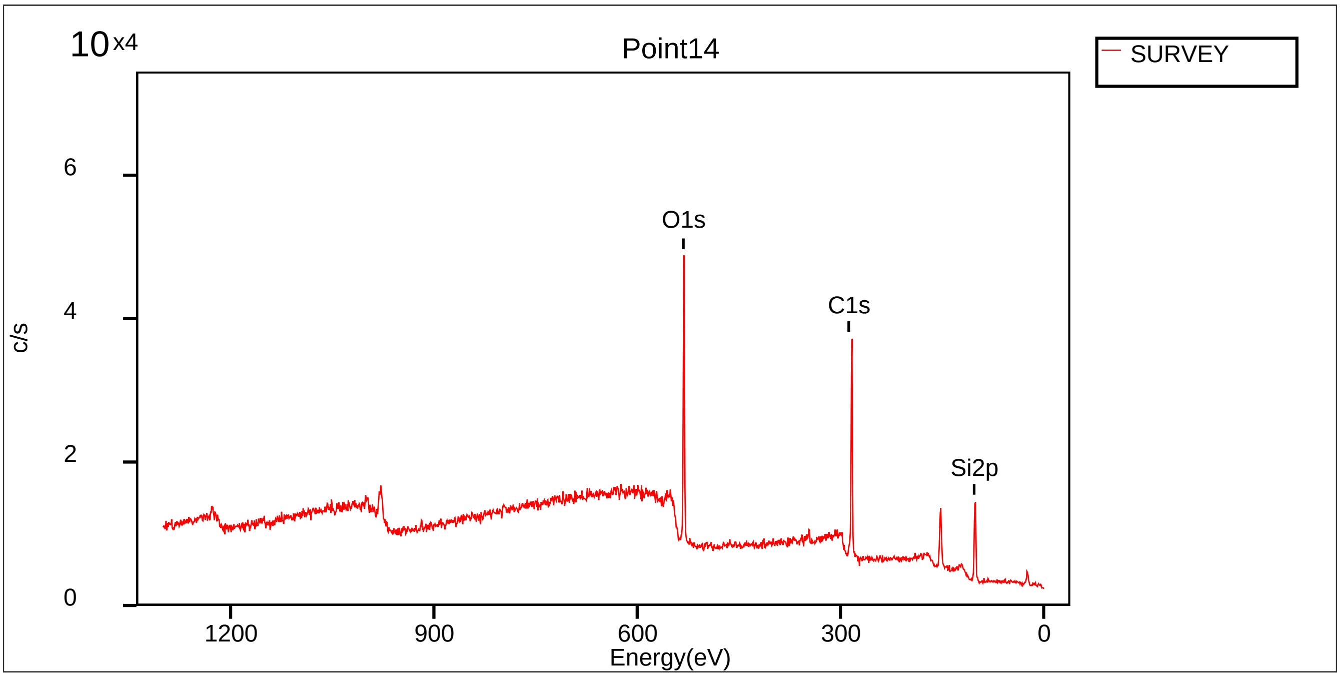

fig. 2.2.14 Survey Point14

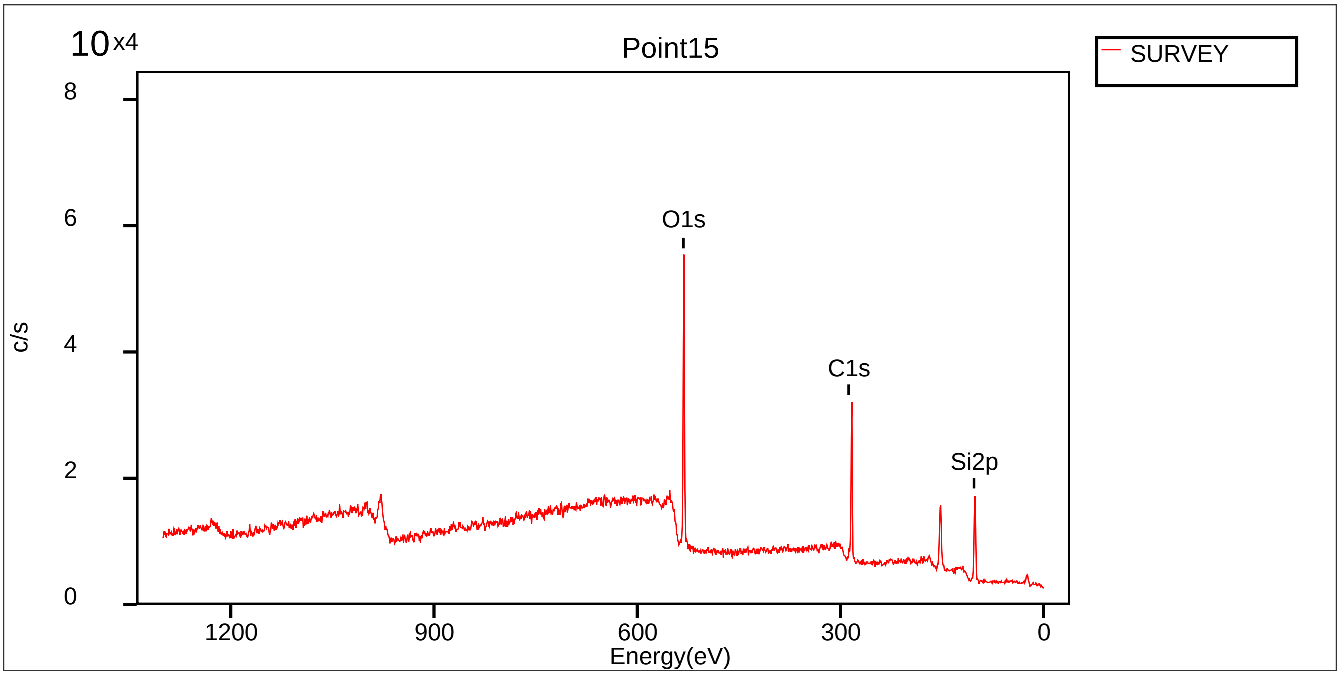

fig. 2.2.15 Survey Point15

2.3 Narrow

table. 2.3.1 File Table

Position	File Path
1	20140604_140102¥20140604 PDMS liao¥14604_142948.spe
2	20140604_140102¥20140604 PDMS liao¥14604_143237.spe
3	20140604_140102¥20140604 PDMS liao¥14604_143622.spe
4	20140604_140102¥20140604 PDMS liao¥14604_144030.spe
5	20140604_140102¥20140604 PDMS liao¥14604_144427.spe
6	20140604_140102¥20140604 PDMS liao¥14604_144812.spe
7	20140604_140102¥20140604 PDMS liao¥14604_145213.spe
8	20140604_140102¥20140604 PDMS liao¥14604_145449.spe
9	20140604_140102¥20140604 PDMS liao¥14604_145757.spe
10	20140604_140102¥20140604 PDMS liao¥14604_150156.spe
11	20140604_140102¥20140604 PDMS liao¥14604_150433.spe
12	20140604_140102¥20140604 PDMS liao¥14604_150820.spe
13	20140604_140102¥20140604 PDMS liao¥14604_151217.spe
14	20140604_140102¥20140604 PDMS liao¥14604_151610.spe
15	20140604_140102¥20140604 PDMS liao¥14604_151956.spe

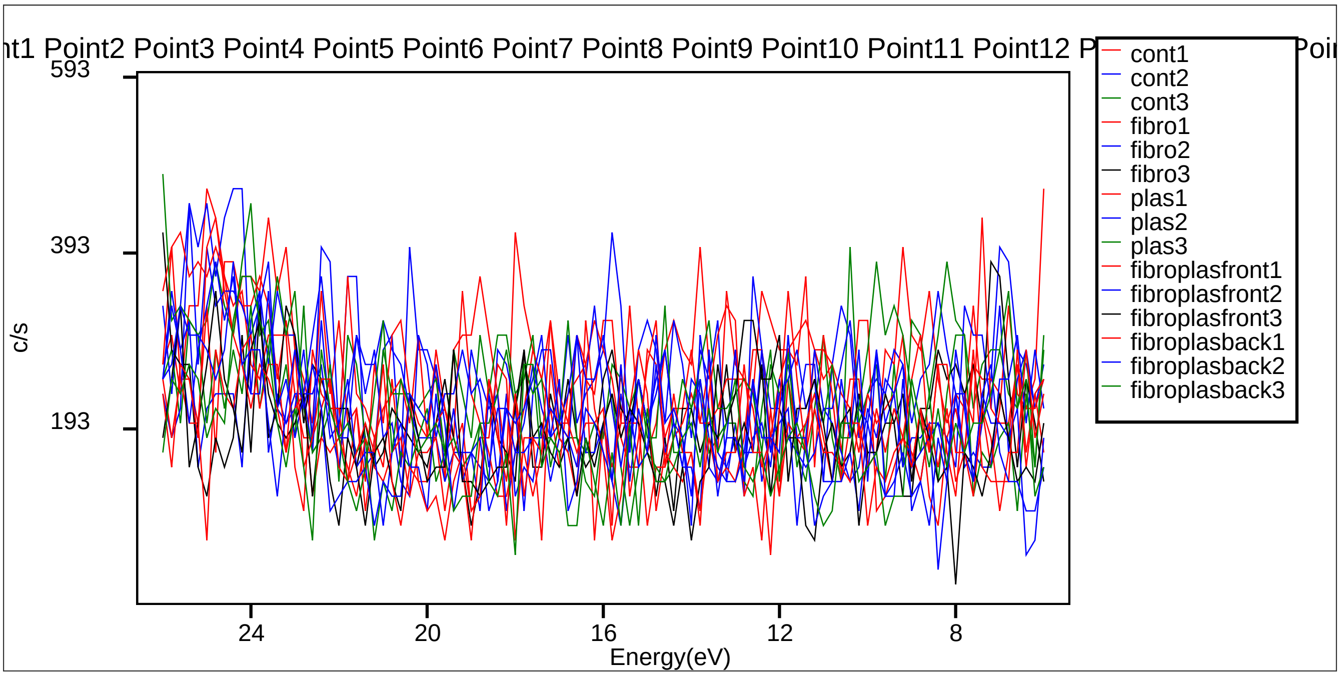

fig. 2.3.1 H1s

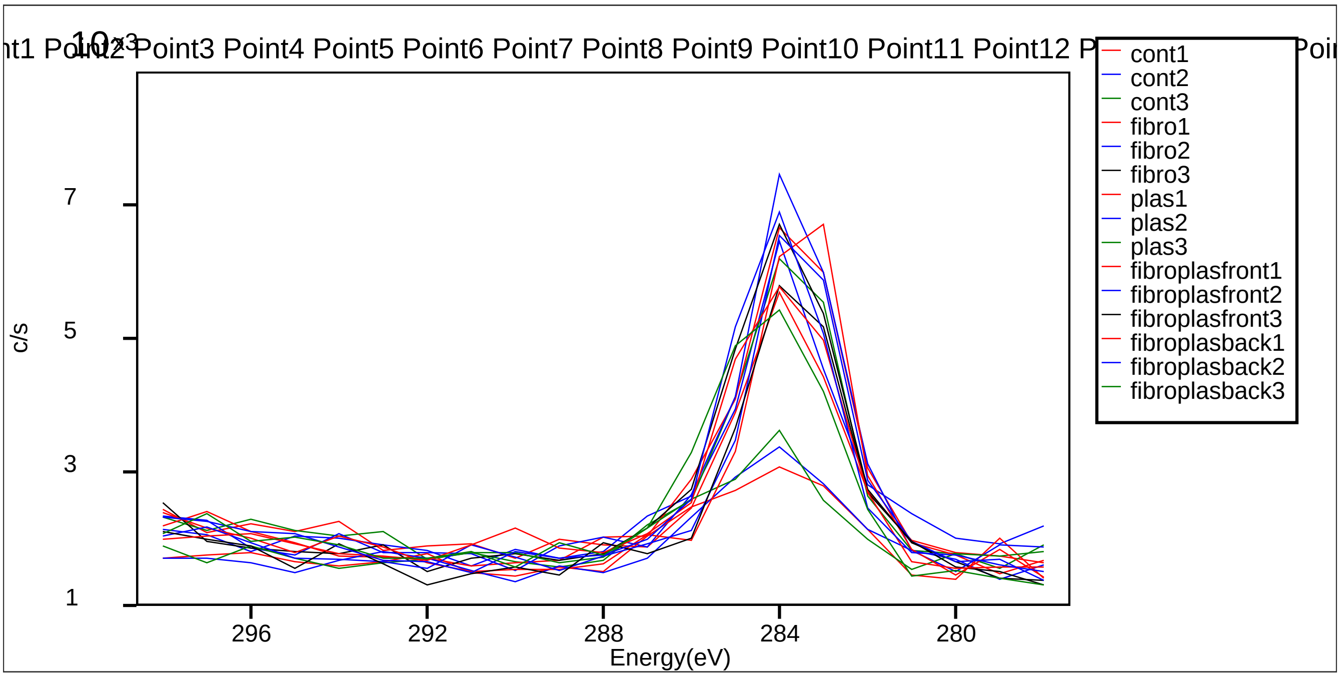

fig. 2.3.2 C1s

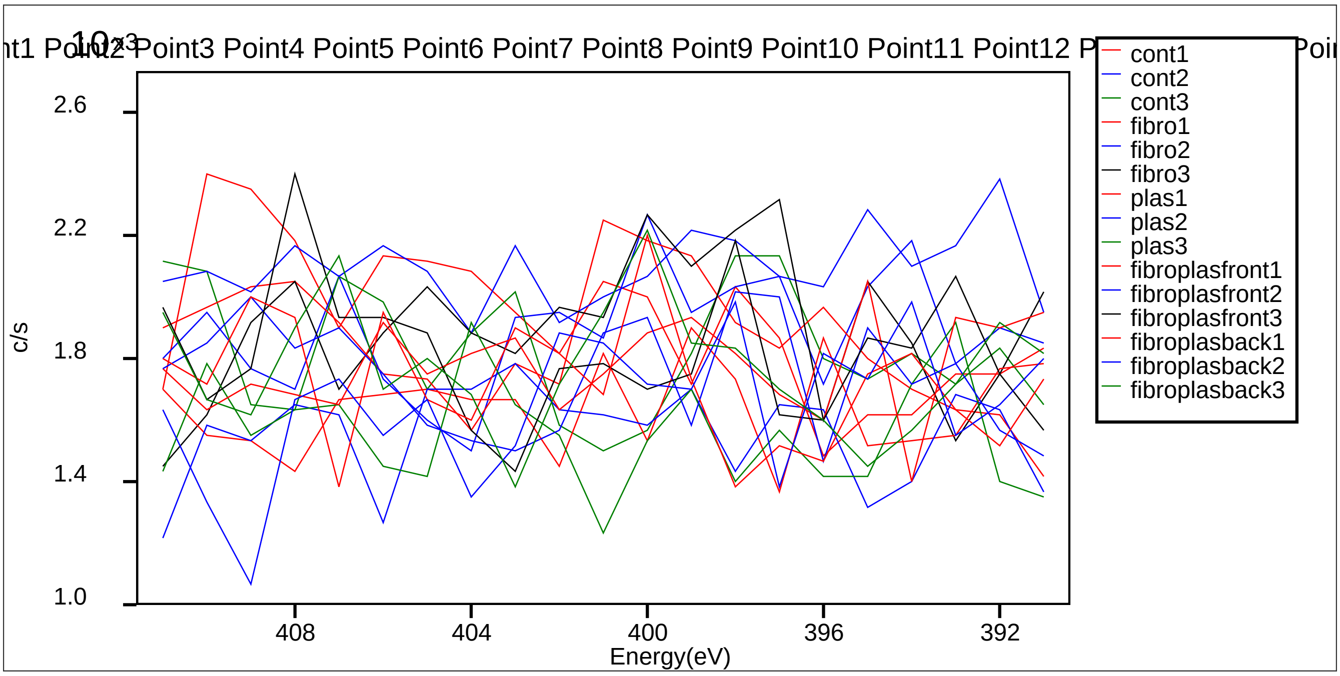

fig. 2.3.3 N1s

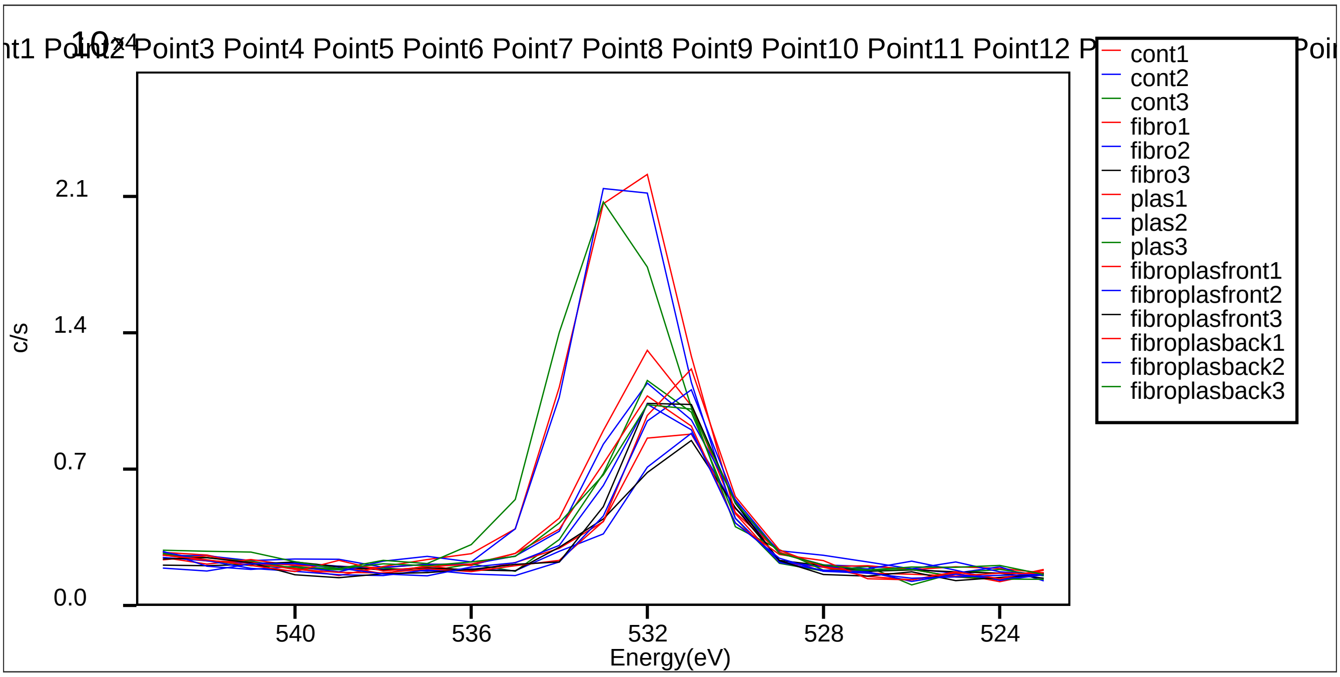

fig. 2.3.4 O1s

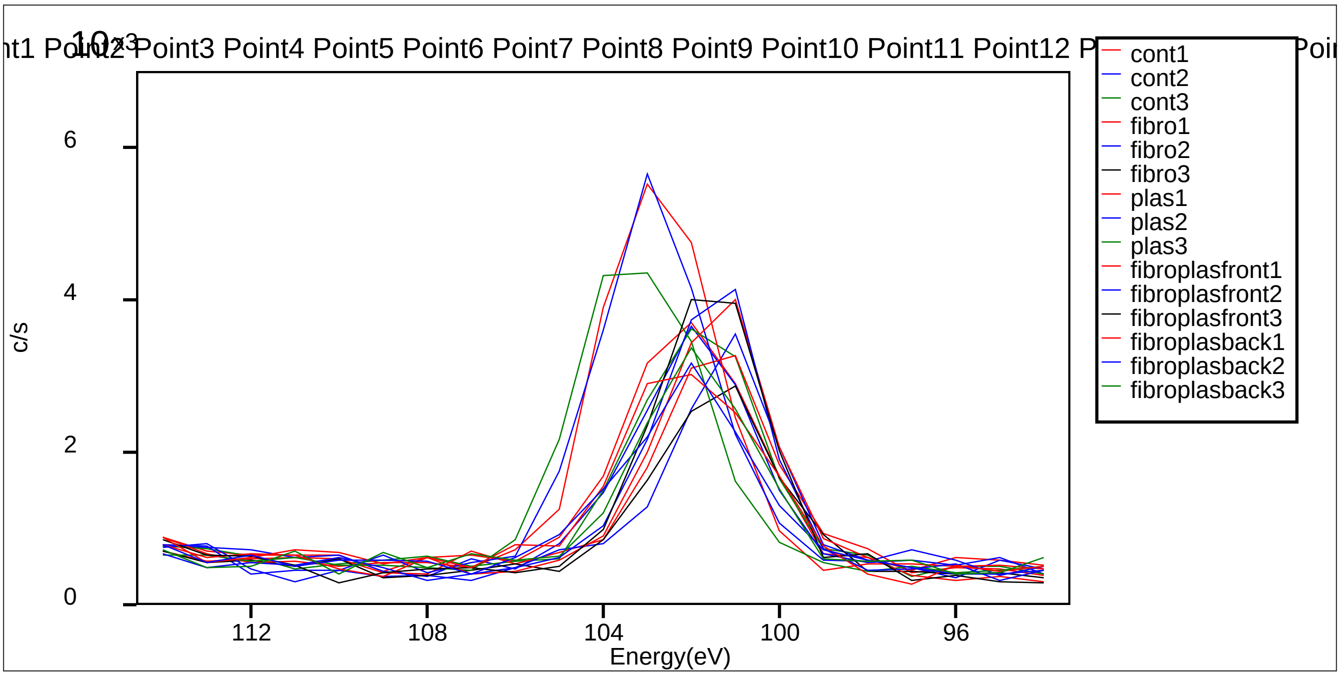

fig. 2.3.5 Si2p

3. Memo
